# Valorization of domestic wastes into Cu-MOF-derived CuO@ZnO nanocomposites for sustainable photocatalytic degradation of methylene blue and rhodamine B dyes

**DOI:** 10.1038/s41598-026-51864-6

**Published:** 2026-05-13

**Authors:** M. S. Samy, H. M. Abou El Nadar, E. A. Gomaa, Amr Awad Ibrahim, Mina Shawky Adly

**Affiliations:** 1 Department of Chemistry, Faculty of Science, Mansoura University, Al-Mansoura, 35516 Egypt; 2 Energy & Desalination Center, Faculty of Science, Mansoura University, Mansoura, Egypt

**Keywords:** Photocatalytic degradation, Metal–organic frameworks, Waste PET, Mott-Schottky, Chemistry, Environmental sciences, Materials science, Nanoscience and technology

## Abstract

The conversion of waste materials into value-added functional photocatalysts represents a sustainable strategy for environmental restoration. Metal–organic frameworks (Cu-BDC) synthesized from waste-derived precursors were utilized as templates to construct CuO@ZnO n–n heterojunction photocatalysts. XRD analysis confirmed the growth of wurtzite ZnO and monoclinic CuO phases, as evidenced by selected-area electron diffraction (SAED) patterns showing well-defined diffraction rings, indicating crystallinity. HRTEM revealed lattice spacings of 0.262 nm and 0.232 nm corresponding to ZnO (002) and CuO (111), respectively. DRS-Tauc analysis demonstrated enhanced visible-light absorption and a narrowing of the bandgap from 3.1 to 2.89 eV upon CuO incorporation. CuO_0.25_@ZnO achieved the highest photocatalytic performance, with 99.0% and 97.7% degradation of methylene blue (MB) and rhodamine B (RhB), respectively. The degradation kinetics followed pseudo-first-order kinetics, with rate constants of 0.025 and 0.024 min^−1^ for MB and RhB, respectively, which were higher than those for pure ZnO. Photoelectrochemical measurements and photoluminescence (PL) spectra revealed high charge separation and reduced recombination of CuO_0.25_@ZnO. Scavenger experiments and terephthalic acid fluorescence analysis confirmed the generation of reactive species, supporting a Z-scheme charge-transfer mechanism. Ultimately, we anticipate that this novel photocatalyst will have meaningful applications in the energy sector and environmental remediation.

**Supplementary Information:**

The online version contains supplementary material available at 10.1038/s41598-026-51864-6.

## Introduction

Water supplies must remain intact for humans to survive and for Earth’s ecosystems to thrive. In recent decades, industrial growth and evolving human lifestyles have led to various health issues caused by water and air pollution^1,2^. The wide use of dyes in the printing, leather, and textile industries has raised significant environmental and health concerns^3,4^. It is estimated that about 20% of textile dyes are released as wastewater, dyeing and textile effluents account for 17–20% of global water pollution^5^. These dyes are non-biodegradable and carcinogenic, posing serious health risks to humans and aquatic ecosystems^6^. In particular, rhodamine B (RhB) dye, a water-soluble cationic xanthene dye, can irritate the skin, eyes, and respiratory tract and exhibit carcinogenic and neurotoxic effects^7^. According to recent survey results, about 65% of the world’s population will be adversely affected by freshwater scarcity by 2050^8,9^. These dye effluents are degraded naturally by sunlight, but the process is slow^10^. The traditional methods for reducing dye contamination, such as ozonation, chlorination, and membrane filtration, are ineffective due to their high operating costs, prolonged treatment times, and secondary sludge formation^11,12^. Nowadays, chemical methods, including advanced oxidation processes (AOPs) such as photocatalysis, are more effective. It is highly efficient, low-cost, and environmentally friendly, with high reaction rates that are not time-consuming, and it does not produce secondary pollution^13^. That is dependent on the presence of a semiconductor material, which produces electron–hole pairs (${\mathrm{e}}^{ - } /{\mathrm{h}}^{ + }$), that react with water and dissolved oxygen to produce reactive oxygen species (ROS). These species include hydroxyl radicals (HO^•^) and superoxide radical anions $\left( {{\mathrm{O}}_{2}^{ \bullet - } } \right)$ to oxidize and degrade organic and inorganic pollutants in water without toxic byproducts, thereby ensuring their success^14–20^. Additionally, adding activators such as sodium borohydride (NaBH_4_) and peroxymonosulfate (PMS) can improve the breakdown of organic contaminants by the formation of ${\mathrm{SO}}_{4}^{ - }$ and ${\mathrm{HO}}^{ \bullet }$ radicals that target pollutants^21,22^. Photocatalysis has become a highly promising and adaptable technique that has attracted considerable attention across a variety of fields, including wastewater treatment, owing to its efficiency and sustainability. It has shown remarkable potential for applications like hydrogen production, CO_2_ reduction, and microbial disinfection^23,24^. Furthermore, the photo-Fenton process is important for pollutant removal, as it generates reactive hydroxyl radicals via ferrous ions and H_2_O_2_ under visible light, thereby facilitating pollutant degradation^25,26^.

Semiconductor nanostructures based on metal oxides, including ZnO, CuO, Cu_2_O, TiO_2_, BiVO_4_, WO_3_, and NiO, have superior photocatalytic degradation capabilities and improve incident photon absorption^27–32^. ZnO and TiO_2_ nanoparticles have similar valence and conduction bands, as well as similar electron affinities and energy levels^33,34^. Because ZnO absorbs a larger fraction of the solar spectrum than TiO_2_, it has become increasingly popular recently due to its simple fabrication, affordability, environmental sustainability, and improved photocatalytic activity^30,35^. However, ZnO has many disadvantages, including a wide band gap of approximately 3.2 eV that impedes electron and hole transport to the surface for reaction and increases the recombination rate. It also absorbs in the UV region (λ = 380 nm) with only 5% absorption in the visible spectrum^36–39^. Enhancing photocatalytic efficiency largely depends on suppressing the recombination of electron–hole pairs. Therefore, understanding and regulating the recombination mechanism is essential for improving photocatalyst performance. Various strategies have been adopted to inhibit charge recombination, including doping ZnO with non-metals, transition metals, and constructing semiconductor heterojunctions^40,41^. Heterojunction formation using low-bandgap CuO semiconductors (1.2–2.1 eV) is an effective method to overcome ZnO’s low photodegradation efficiency by extending its absorption into the visible. It also enhances charge separation of photogenerated electron–hole pairs, reduces charge-carrier recombination, and increases surface area via additional pore sites, thereby maximizing photodegradation activity^42–44^. Moreover, CuO@ZnO nanocomposites have already been proposed for various applications, including photocatalytic activity, magnetic properties, conductivity studies, and gas sensing^45,46^.

Metal oxides with well-controlled morphologies can be synthesized via direct air annealing of MOFs, yielding materials with higher surface areas and optimal pore structures^47^. Doustkhah et al. demonstrated that nanocrystalline ZnO with controlled orientation generated from MOF-5 exhibits excellent photocatalytic degradation of MB dye, demonstrating the superiority of MOF-derived metal oxides^48^. One of the main costs of MOF production is the use of linkers; therefore, the recycling of polyethylene terephthalate (PET) bottles using a chemical method is considered a green chemical recycling approach and a cost-effective source of terephthalic acid, contributing to the elimination of PET waste and leading to a cleaner environment^49,50^.

PET bottles are among the most common waste materials globally, owing to their extensive use as packaging materials for their high resistance to moisture, sunlight, and microorganisms, their transparent design, structural versatility, inertness toward food, and affordability. In fact, the estimated annual worldwide consumption of PET is about 24 million tons and is steadily increasing. Because PET is non-biodegradable, it causes significant environmental problems. Nonetheless, the key element here is the application of green chemical recycling to create high-value products from inexpensive, recyclable materials, fulfilling the waste-to-value-added product principle^51^. Additionally, metal ions (e.g., copper) are obtained via chemical recycling of electrical copper wires.

In this study, Cu-BDC frameworks were successfully fabricated via a green route using recycled polyethylene terephthalate (PET) waste as the organic linker source and copper electrical wire as the metal precursor, making the process low-cost, eco-friendly, and nonhazardous. As a result, CuO nanoparticles were produced from the Cu-BDC MOF via cost-effective recycling of waste materials, promoting community waste recycling. Furthermore, CuO@ZnO nanocomposites were successfully synthesized with different ratios of CuO to study their effect on the optimization of photocatalytic degradation performance of the nanocomposites via a solvothermal method. Analytical techniques were used to elucidate the structure and morphology of the fabricated photocatalysts. Degradation experiments of MB and RhB dyes were examined under UV–visible light to assess the photocatalytic capability of CuO@ZnO in comparison to pure ZnO. Moreover, the photodegradation of colored pollutants at room temperature was examined in relation to the solution’s pH, catalyst dose, and dye concentrations. In addition to radical scavengers, A PL analysis of terephthalic acid was conducted to ascertain the type of ROS involved in the photocatalytic degradation pathway. Finally, photoelectrochemical measurements were employed to assess the photoresponse of the photocatalysts.

## Experimental

### Materials

Zinc acetate dihydrate (Zn(Ac)_2_· 2H_2_O, 98%), cetyltrimethylammonium bromide (CTAB, 98%), methanol (99.5%), N, N-Dimethylformamide (DMF, 99%), ammonium hydroxide (NH_4_OH, 30%), tert-butyl alcohol (*t*-BuOH, 98%), silver nitrate (AgNO_3_, 99%), benzoquinone (BQ, 98.5%), sodium hydroxide (NaOH, 97%), hydrochloric acid (HCl 98%), sodium sulphate (Na_2_SO_4_, 99%), ethylenediaminetetraacetic acid disodium salt (EDTA-2Na, 99.9%), potassium hydroxide (KOH, 99.9%), nitric acid (HNO_3_, 70%), ethylene glycol (C_2_H_6_O_2_, 99.8%), MB and RhB dyes were acquired from Loba Chemie and used without any additional purification. All aqueous solutions utilized in the synthesis and photocatalytic degradation processes were fabricated with distilled water.

### Preparation of ZnO nanoparticles

ZnO was prepared by dissolving 0.5 g of CTAB as a capping agent in 50 mL of a water–ethanol mixture at a ratio of 4:1. Subsequently, 2.697 g of Zn(Ac)_2_. 2H_2_O was added to the prior solution and mixed until a clear solution formed. After that, ammonia was added dropwise with continuous stirring until complete precipitation of Zn^2+^ ions, and the solution pH was adjusted to 9. The white precipitate was segregated and repeatedly washed with distilled H_2_O, then with ethanol. The collected precipitate was left to dry overnight in a conventional oven, then calcined for 3 h at 550 °C with a 5 °C min^−1^ temperature rate^52,53^.

### Preparation of terephthalic acid (BDC)

A hydrothermal process was used to prepare BDC from PET waste, as reported with simple modifications^54,55^. Firstly, the PET plastic (bottles or cups) is washed with distilled H_2_O and cut into smaller pieces. Subsequently, 5.0 g of cleaned PET flakes were placed into a Teflon tube containing 40 mL of distilled water and 5 mL of ethylene glycol, and the mixture was maintained in a conventional oven at 210 ºC for 16 h. After that, the product was centrifuged off and stirred with NaOH (1.5 M) till a clear solution. After that, additional filtration was performed to isolate impurities and unreacted PET flakes, followed by the addition of HCl solution (50% v/v) to obtain terephthalic acid as a white solid. The final white precipitate was washed repeatedly with distilled H_2_O until neutral pH and dried at 80 °C for 24 h^56^.

### Extraction of Cu(NO_3_)_2_ metal from electrical copper wires

Clean copper wires (5.0 g) were digested in HNO_3_ (50%) solution and kept for 24 h. The clear blue solution was filtered to remove any residue, then vacuum-dried to form a blue slurry. The resulting copper nitrate solution was kept in a desiccator under vacuum for several days till the formation of blue copper nitrate crystals. Finally, the blue crystals were transferred to a sealed tube for further use.

### Preparation of Cu-BDC(W) and CuO@ZnO composite based Cu-BDC(W)

Cu-BDC was synthesized using equimolar quantities of synthesized copper nitrate (0.526 g) and BDC (0.362 g) dissolved in a 30 mL DMF-methanol mixture with a ratio of 1:1. Then, the previous solution was put into an autoclave reactor at 100 °C for 24 h. After centrifuging and repeatedly washing the light-blue precipitate with DMF and methanol, it was vacuum-dried overnight, yielding approximately 0.536 g^57^.

For the preparation of CuO@ZnO, 1.0 g of ZnO was sonicated for 15 min in a mixture of DMF and methanol till the formation of a homogeneous suspension. Subsequently, 0.362 g of BDC and 0.526 g of Cu(NO_3_)_2_ solution were added to the ZnO suspension, which was then transferred to an autoclave reactor and heated at 100 ºC for 24 h. The collected powder was calcined at 550 °C for 3 h under air. The concentrations of Cu-BDC loaded on ZnO nanoparticles were adjusted to 12.5, 25, and 50.0 wt%, and the fabricated nanocomposites were defined as CuO_0.12_@ZnO, CuO_0.25_@ZnO, and CuO_0.50_@ZnO nanocomposites, respectively.

### Photocatalytic Activity

The activity of the fabricated photocatalysts was assessed by photocatalytic degradation of MB and RhB dyes at room temperature. The photocatalytic experiment was carried out using 1.0 g L^−1^ of photocatalyst, ultrasonically dispersed in a 50 mL dye solution at 10 mg L^−1^, at neutral pH. The photocatalyst with the dye mixture was agitated for 30 min in the dark to achieve adsorption/desorption equilibrium. A 250 W mercury lamp was then placed 20 cm from the reaction mixture, and 1 mL of the solution was withdrawn at 15 min intervals. The aliquots were diluted with distilled H_2_O and centrifuged to obtain a clear supernatant. The absorbance values of the remaining dye samples were estimated by UV–visible spectrophotometry at 554 and 665 nm for RhB and MB dyes, respectively. The efficiencies of the prepared photocatalysts were calculated according to the following expression^58^:

1 $${\text{Degradation }}\left( {{\% }} \right) = \left( {1 - \frac{{\mathrm{C}}}{{{\mathrm{C}}_{{\mathrm{o}}} }}} \right) \times 100$$

where; C_o_ and C refer to the concentrations before and after illumination under UV–visible light.

### Electrochemical measurements

The electrochemical experiments were processed in a quartz cell using 0.5 M Na_2_SO_4_ as electrolyte to facilitate charge transfer in a three-electrode setup. The drop-casting technique was used to establish an indium-tin oxide (ITO) glass working electrode, and the reference and auxiliary electrodes were silver/silver chloride and a platinum sheet, respectively. Generally, 1 mL of ethanol solution was used to sonicate 5 mg of the fabricated photocatalyst for 1 h at ambient temperature. Then, 10 μL of the prepared suspension was coated onto 1 $\times$ 1 cm^2^ of ITO glass to form a uniform layer of the photocatalyst. The constructed ITO working electrode was air-dried before use in electrochemical measurements. CS Studio 6 software was used to control the three electrodes, which were connected to the Corrtest series CS315 workstation 6.2. The photoresponse of the prepared photocatalysts was measured under visible light, with the light turned on and off for 600 s. The light source irradiation was a 150 W Xe lamp. Additionally, electrochemical impedance spectroscopy (EIS) was measured in 0.1 M KOH as the electrolyte at the open-circuit potential (OCP) over a frequency range of 0.01–10^5^ Hz. Finally, the Mott-Schottky (MS) test was conducted from − 1 to 1 V with a scan rate of 50 mV s^−1^ at a frequency of 5 kHz.

### Characterization

The morphologies and element percentages of the fabricated nanocomposites were examined using scanning electron microscopy (SEM, JEOL-JSM-6510 LV) with EDX analysis. The sample was sputter-coated with a thin coating of gold before SEM analysis and transmission electron microscopy (TEM) with a JEOL-JEM-2100. N_2_ adsorption/desorption isotherms of the photocatalysts were conducted on BELSORP MINI X (MICROTRAK Co., Japan) to measure the pore structures, and the surface area of the catalyst was calculated by Barrette-Joyner-Halenda (BJH) and Brunauer–Emmett–Teller (BET) methods, respectively. Before the analysis, the photocatalysts were degassed at 150 °C for 120 min. FT-IR spectroscopy in the 4000–400 cm^−1^ range was used to analyze the functional groups in the resulting photocatalysts with a Mattson FT-IR-5000S spectrophotometer equipped with ATR. X-ray diffraction (XRD) patterns were employed at a 2θ range of 10°−80° using monochromated Cu Kα radiation. The absorbance of colored materials and organic dyes was measured using the Peak Instrumentation (C-7200) UV–vis spectrophotometer. Thermogravimetric analysis (TGA) was performed to study the thermal stability of as-synthesized Cu-BDC frameworks using authentic and waste precursors (Shimadzu TGA-50 series) under N_2_ at a heating rate of 10 °C min^−1^. The Electron spin resonance (ESR) signals of the generated free radicals were recorded using an X-band EMX spectrometer (Bruker, Germany) equipped with a standard rectangular cavity (ER 4102) and a microwave power of 0.5 W. The surface compositions of the generated photocatalysts and the oxidation states of their constituent elements were investigated using X-ray photoelectron spectroscopy (XPS) with a Thermo Fisher Scientific monochromatic Al Kα X-ray source. The PL spectra were examined using a SHIMADZU RF-5301PC spectrofluorophotometer with a 150 W Xe lamp, and the diffuse reflectance spectra (DRS) were collected using a Jasco spectrophotometer, coupled with a reflectivity measurement covering 220–2000 nm, which was used to measure the diffusely reflected light and absorption spectra in the UV–visible region.

## Results and discussion

### Characterization

**Fig. 1:**
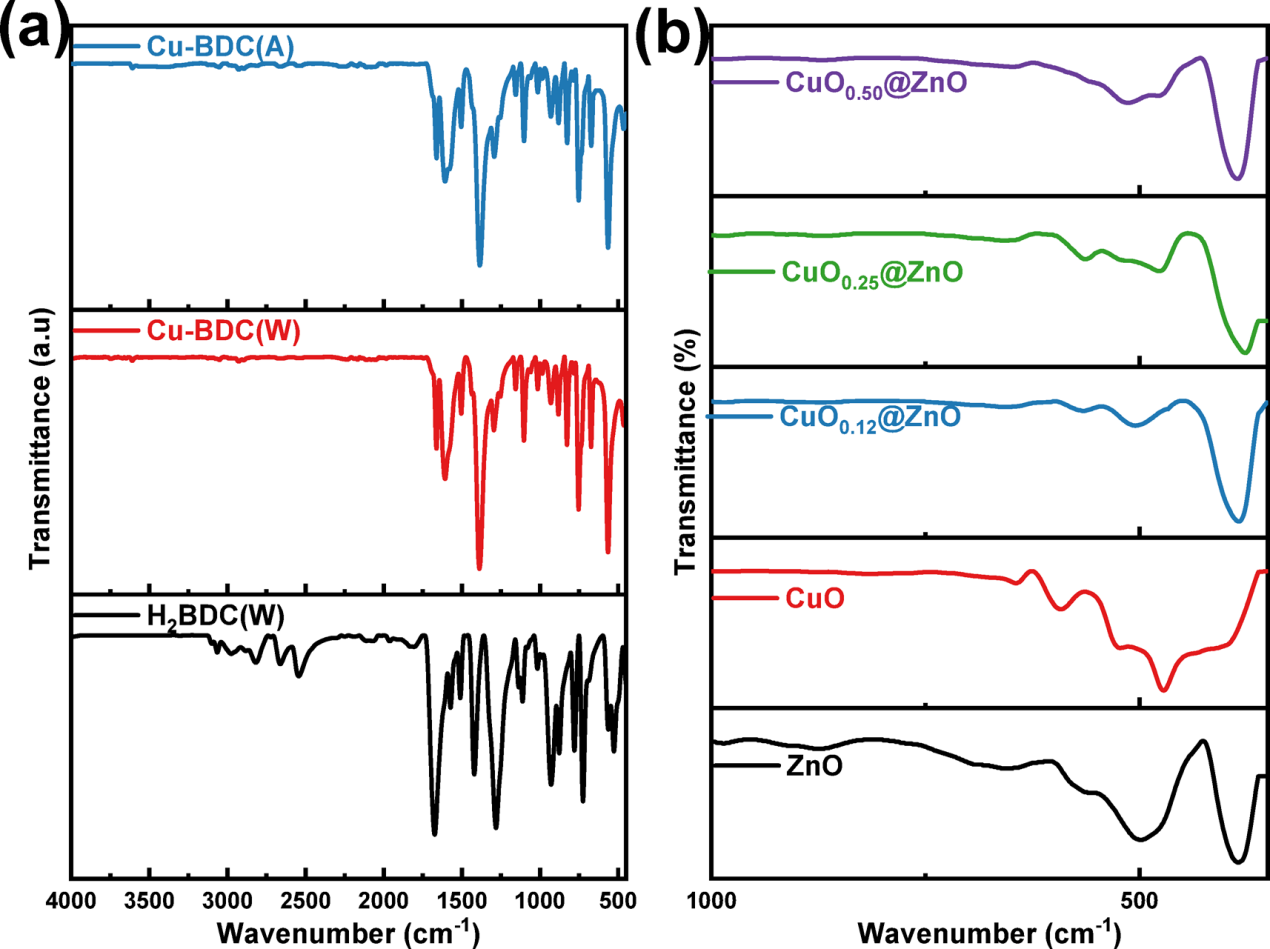
FT-IR spectra of (**a**) H_2_BDC and Cu-BDC samples synthesized from wastes and authentic materials, (**b**) pure CuO, ZnO, and CuO@ZnO nanocomposites of different ratios.

As shown in Fig. 1a, FTIR spectroscopy was performed to confirm the successful synthesis of the terephthalic acid solid and to identify the functional groups in the as-prepared photocatalysts. The bands in the range 2814–2550 cm^−1^ correspond to the stretching vibration of O $-$ H in the carboxylic acid group. The appearance of a band at 3065 cm^−1^ indicates aromatic C $-$ H stretching vibrations. The stretching vibration of C = O in the carboxylate group is demonstrated by the band at 1674 cm^−1^. The band observed at 1281 cm^−1^, which refers to the C $-$ O bond stretching vibration in the carboxylic acid group. The band at 730 cm^−1^ indicates the C-H bending vibration^59,60^. The absorption bands observed in the range of 1572–1509 cm^−1^ are attributed to stretching vibrations of aromatic C = C. The FTIR spectrum more closely matched previously reported data, indicating the successful preparation of terephthalic acid from PET plastic waste via hydrolysis. FT-IR spectra of Cu-BDC using authentic and waste materials showed highly intense and clear bands at 1382 cm^−1^ and 1603 cm^−1^, which are related to the coordinated carboxylate groups’ stretching modes in symmetric (*v*_s_) and asymmetric (*v*_as_) positions, respectively. The aromatic rings are characterized by narrow and weak bands at 665 and 1100 cm^−1^ assigned to the vibrations of γ(C–H) and δ(C–H), respectively, demonstrating the presence of the BDC linker. The vibrations of the phenyl ring are characterized by the two bands allocated at 1508 and 751 cm^−1^. The absorption bands at 466 and 561 cm^−1^ correspond to Cu–O stretching vibrations^61–63^. The carbonyl group in DMF is responsible for the band at 1666 cm^−1^^64^. Both synthesized authentic and waste Cu-BDC frameworks exhibited the same bands as illustrated in Fig. 1a. The effective synthesis of Zn–O and Cu–O in each specimen was verified by the existence of bands ranging from 400 to 600 cm^−1^. The band at 508 cm^−1^ is ascribed to Zn $-$ O stretching vibrations. Further, in the CuO@ZnO nanocomposites spectrum, the peak intensity of Zn $-$ O was reduced upon the incorporation of CuO, which proves the preparation of CuO@ZnO composites, as shown in Fig. 1b^65^.

**Fig. 2:**
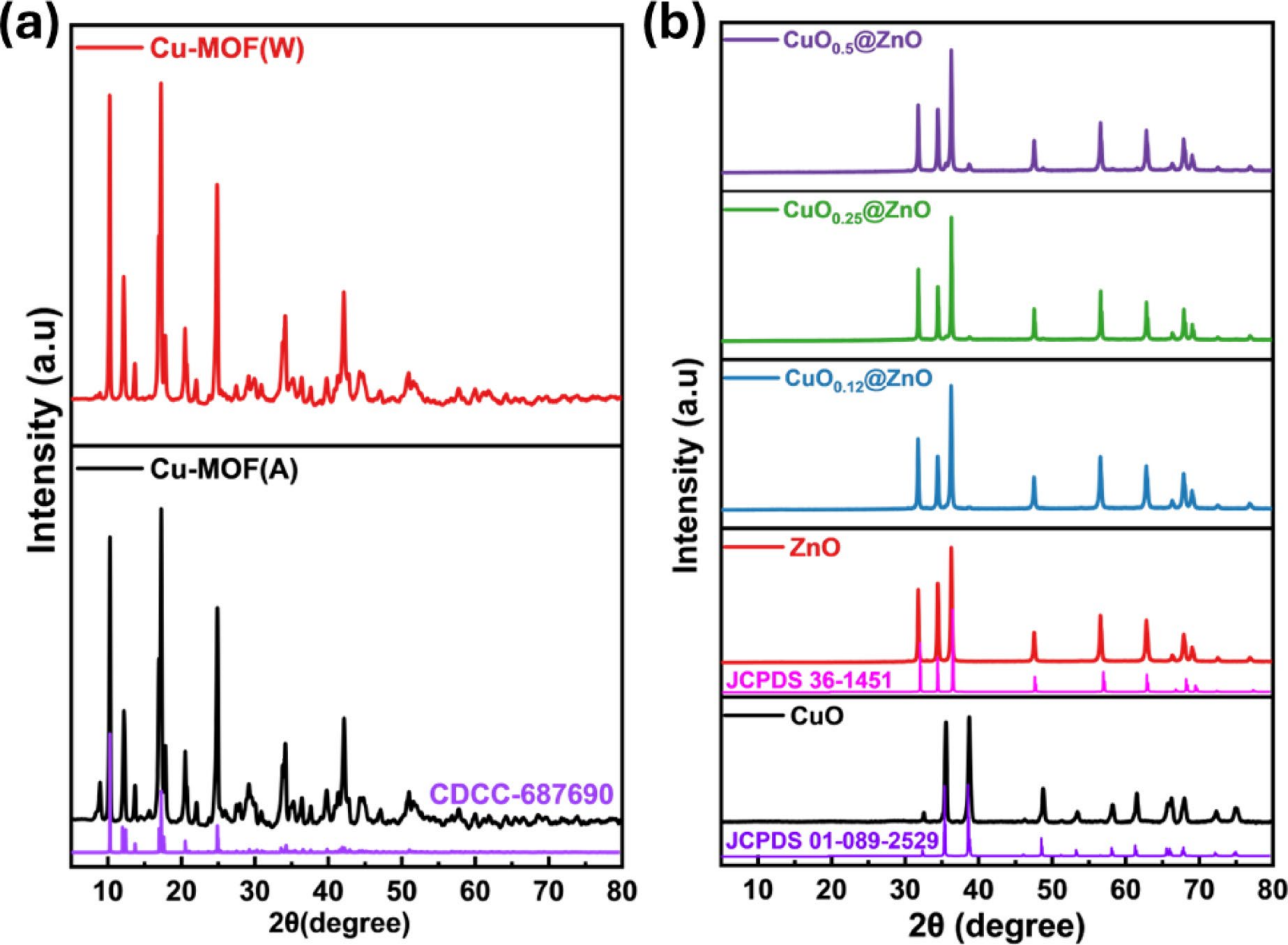
XRD patterns (**a**) of Cu-BDC MOFs, (**b**) pure CuO, ZnO, and different ratios of CuO@ZnO nanocomposites.

The crystal phase of the as-prepared photocatalysts was characterized by XRD. The patterns of both Cu-BDCs prepared from authentic and waste materials are identical (Fig. 2a). These results provide convincing evidence for the practical synthesis of Cu-BDC frameworks based on metal nodes and organic linkers derived from domestic waste. The Cu-BDC framework patterns exhibit several distinct peaks of high intensity. This illustrates the exceptional crystallinity of the generated frameworks, which was observed at 2θ $=$ 10.2°, 12.3°, 13.6°, 17.4°, 20.6°, 24.9°, 29.3°, 34.0°, and 42.2° were assigned to crystal planes of (110), (020), (11 $\overline{1 }$), ($\overline{2 }$ 01), (111), (220), (131), (31 $\overline{2 }$), and ($\overline{4 }$ 02) (CDCC-687690). According to the previous related reports, these results confirm that MOFs crystallize in monoclinic symmetrical structures^59,66–69^. The XRD pattern of pure ZnO provides distinctive peaks positioned at 31.8°, 34.5°, 36.4°, 47.6°, 56.7°, 62.6°, 66.3°, 67.9°, and 69.5° that indexed as (100), (002), (101), (102), (110), (103), (200), (112), and (201) planes, respectively^70^. A good agreement is observed between the crystalline diffraction data and the standard hexagonal wurtzite ZnO patterns (JCPDS 36–1451)^71,72^. Moreover, the pattern of CuO nanoparticles displayed diffraction peaks at 32.6°, 35.6°, 38.8°, 49.0°, 53.5°, 58.3°, 61.5°, 66.3°, 67.9°, 72.7°, and 75.1° corresponding to (110), (11 $\overline{1 }$), (111), (20 $\overline{2 }$), (020), (202), (11 $\overline{3 }$), (022), (31 $\overline{1 }$), (220), and (311) planes, respectively, which are ascribed to the monoclinic phase of CuO that correspond closely with (JCPDS 01–089–2529)^73^. Further, the CuO_0.25_@ZnO nanocomposite shows a very low intensity of CuO because of the low quantity of CuO nanoparticles loaded on the ZnO nanoparticles, which does not appear in the XRD pattern^74^. The diffraction pattern of the fabricated nanocomposites showed low CuO intensity at lower concentrations, which increased with higher CuO levels. The characteristic peaks related to ZnO are not affected in all composites with different loading percentages of CuO, which indicates the successful formation of CuO@ZnO^65^. Lack of additional peaks in the XRD patterns further suggests that the photocatalyst materials are impurity-free. As seen in Fig. 2b, the strong peaks also indicate high crystallinity and the absence of other phases in the nanocomposites. The Scherrer equation in Eq. (2) is used to calculate the crystal sizes of the prepared materials as follows:

2 $$D = \frac{{{\rm K}\lambda }}{\beta \cos \theta }$$

where; K is the Scherrer constant equals to 0.94, $\lambda$ is the wavelength of the X-ray beam used (0.15406 nm), $\beta$ is the full width at half maximum (FWHM) of the peak, and $\theta$ is the Bragg angle^75^. The average crystallite sizes of CuO, ZnO, and CuO_0.25_@ZnO are noted to be 35.2, 56.3, and 61.4 nm, respectively, and the crystallite sizes of all composites are presented in Table S1.

The surface areas of ZnO NPs and CuO_0.25_@ZnO, determined from N_2_ adsorption–desorption isotherms, are illustrated in Fig. S1. Understanding the variables that affect photocatalytic performance is essential, especially dye adsorption on catalyst surfaces. In the nanocomposite, the adsorption–desorption behavior displays a type II isotherm with H3 hysteresis loop, known as a slit pore structure^76^. The surface area of ZnO NPs and CuO_0.25_@ZnO was determined to be 5.1 and 3.98 m^2^ g^−1^, respectively. After the incorporation of CuO onto the ZnO, the surface area decreased due to the strong deposition of CuO onto the ZnO surface, forming CuO_0.25_@ZnO heterojunction, matched with XRD results, which demonstrates an increase in crystal size of CuO_0.25_@ZnO compared to ZnO^77^. Moreover, the porosity of ZnO NPs and CuO_0.25_@ZnO was measured using BJH pore size analysis, which showed pore sizes of 1.1 nm and 2.8 nm, respectively. These results suggest that CuO_0.25_@ZnO exhibits mesoporousity, facilitating efficient mass transport and the diffusion of dye molecules toward active sites, thereby enhancing photocatalytic efficiency compared to ZnO NPs^78^.

**Fig. 3:**
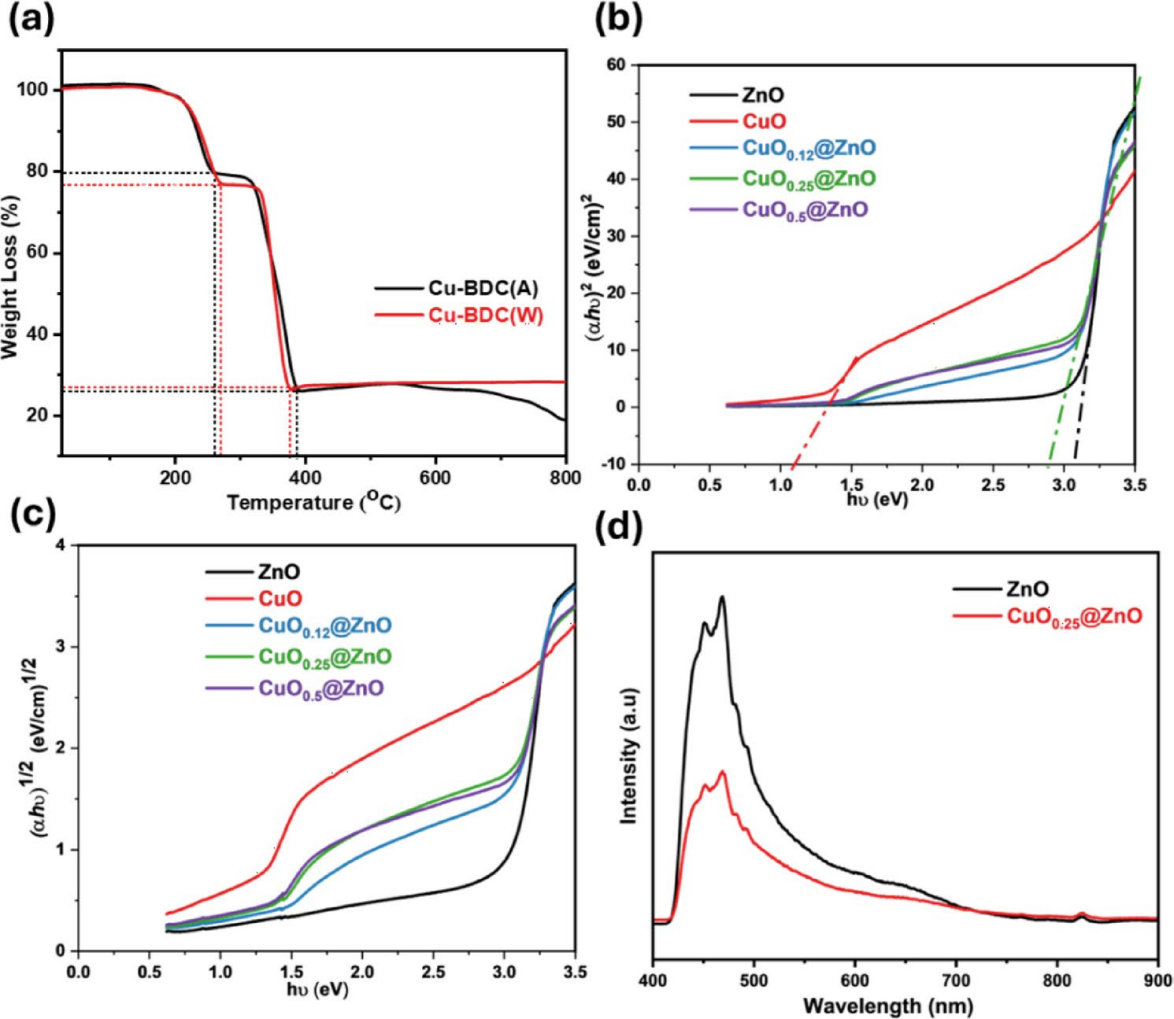
(**a**) TGA analysis, the energy bandgap (Tauc plot) for (**b**) direct and (**c**) indirect transitions of the as-synthesized samples and (**d**) PL spectra of ZnO NPs, and CuO_0.25_@ZnO composite.

The degradation and thermal stability of Cu-BDC(A) and Cu-BDC(W) are described by TGA under N_2_ atmosphere up to 800 °C. The two samples’ decomposition steps are similar, revealing that both MOFs have the same structure with two weight-loss fragments, related to the endothermic process as depicted in Fig. 3a. The first step showed 20% mass loss due to the coordinated DMF molecule in the frameworks at a temperature range of 160–250 °C. Moreover, the second step, from 250 to 385 °C, attributed to the decomposition of the linker and the loss of the BDC^2-^ moiety from Cu-BDC frameworks, corresponds to 54% mass loss^79–81^. Based on the weight calculation, the stable CuO nanoparticles are eventually obtained, and the mole ratio of the Cu^2+^:BDC linker: DMF is approximately 1:1:1, according to the [Cu(BDC)(DMF)] empirical formula^82^.

The optical properties of the photocatalysts were assessed using UV-DRS measurements. The band gap narrowing was quantitatively determined from Kubelka–Munk-transformed DRS data and Tauc plots, rather than relying solely on absorption-edge estimation. The collected spectra of the as-synthesized nanocomposites were examined over 200–800 nm. In contrast to pure ZnO and CuO, the nanocomposite shifts from the ultraviolet to the visible area, as seen in Fig.S2. The band gap energies of fabricated materials were calculated by applying the Tauc formula as indicated in Eq. (3)^83,84^.

3 $$\left( {\alpha h\nu } \right)^{n} = A\left( {h\nu - E_{g} } \right)$$

where; α represents the photocatalyst’s absorption coefficient at a specific wavelength, and A is a constant. ν is the wavenumber, h is the Planck constant, h*ν* is the incident photon energy, E_g_ is the band gap energy, and n (indicating direct and indirect, where n $=$ 2 for direct allowed, n $=$ 1/2 for indirect allowed, n $=$ 2/3 for direct forbidden transitions, and n $=$ 1/3 for indirect forbidden transitions)^78,85^. By applying direct allowed transitions, the bandgap values were calculated as 1.10, 3.10, and 2.89 eV for CuO, ZnO, and CuO_0.25_@ZnO, respectively, as depicted in Fig. 3b. Also, the indirect allowed transitions were obtained as presented in Fig. 3c. The results show a noticeable shift toward lower band gap energy after CuO incorporation, indicating band gap narrowing and improved visible light absorption. ZnO exhibits a wide band gap of approximately 3.1 eV, which limits its photoresponse mainly to the ultraviolet region. Following modification, the bandgap of the CuO_0.25_@ZnO composite decreased to 2.89 eV due to the doping of narrow-bandgap CuO (1.1 eV), which introduces intermediate energy states and broadens light absorption into the visible spectrum. This enhancement facilitates the excitation of additional electrons under visible-light irradiation, increases electron–hole pair generation, promotes effective charge separation, and diminishes electron–hole recombination, thereby improving photocatalytic performance^65^.

PL analysis is used to identify the efficiency of electron–hole separation and recombination in nanomaterials^86^. PL measurements were employed at an excitation wavelength of 380 nm. The PL spectrum of ZnO displayed two blue emission peaks at 450 nm, attributed to zinc vacancies (V_Zn_). In contrast, the 468 nm emission was attributed to the electronic transition between ionized oxygen vacancies and the valence band^87^. As seen in Fig. 3d, by incorporation of CuO nanoparticles into ZnO, the band intensity of the CuO_0.25_@ZnO composite decreases, indicating a decrease in the photoinduced electron–hole pair recombination^88^. Therefore, heterojunction formation significantly enhances the optical properties and photocatalytic efficiency^89^.

**Fig. 4:**
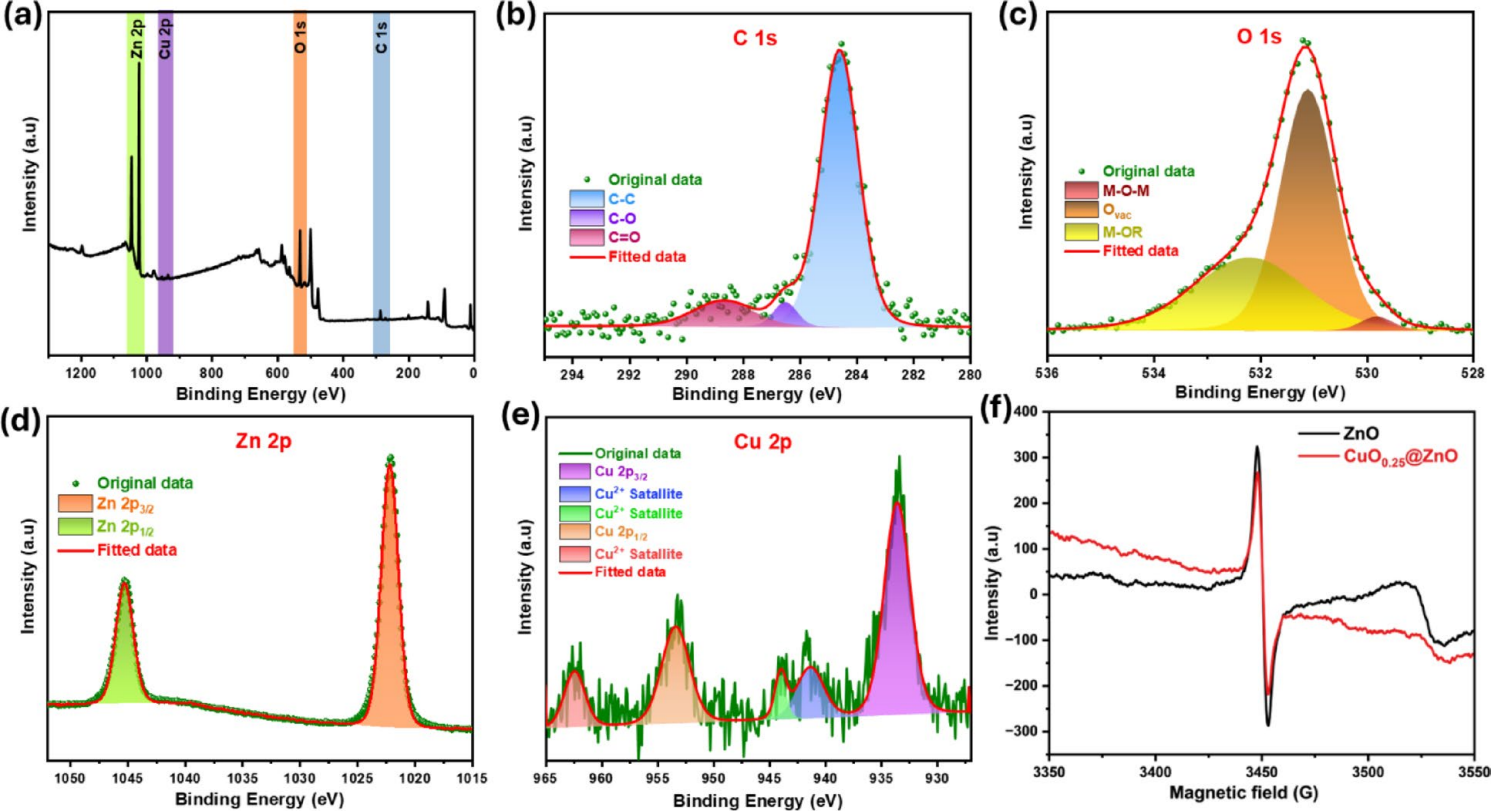
(**a**) XPS survey and HR-XPS patterns (**b**) C 1 s, (**c**) O 1 s, (**d**) Zn 2p and (**e**) Cu 2p of CuO_0.25_@ZnO nanocomposite and (f) ESR spectra of ZnO NPs, and CuO_0.25_@ZnO composite.

XPS was performed to investigate the surface composition of the produced photocatalyst and the oxidation states of its constituent elements. The full survey XPS spectrum of CuO_0.25_@ZnO reveals the existence of C 1 s, O 1 s, Cu 2p, and Zn 2p elements, which confirms the successful preparation as shown in Fig. 4a. As illustrated in Fig. 4b, the HR-XPS spectrum of C 1 s splits into three peaks at 284.5, 286.4, and 288.5 eV, attributed to C $-$ C, C $-$ O, and C $=$ O, respectively^68,90,91^. The HR-XPS spectrum of O 1 s splits into three binding peaks. The M − O − M (M = Cu and Zn) covalent bond is represented by the first peak, which has a binding energy of 530.0 eV. On the other hand, as shown in Fig. 4c, the two other peaks at 531.1 and 532.7 eV are attributed to oxygen vacancies (O_vac_) and weakly bound M-OR species, such as H_2_O, O_2_, and HO^73,92^. The HR-XPS of Zn 2p split into two symmetric peaks at 1022.2 and 1045.2 eV, corresponding to Zn 2p_3/2_ and Zn 2p_1/2_, respectively. As presented in Fig. 4d, the binding energy difference between Zn 2p_1/2_ and Zn 2p_3/2_ is 23.01 eV, indicating that CuO_0.25_@ZnO nanocomposite has a Zn^2+^ oxidation state. Similarly, HR-XPS of Cu 2p was deconvoluted into two prominent peaks at 933.4 and 953.6 eV, corresponding to Cu 2p_3/2_ and Cu 2p_1/2_, respectively. Figure 4e displays two shake-up satellite peaks at 963.0 and 943.1 eV, characteristic of partially filled d-block (3d^9^) Cu^2+^^93,94^. XPS results indicate the presence of Cu^2+^, O^2-^_,_ and Zn^2+^ with well-defined chemical compositions^78^.

ESR is a powerful technique for identifying defect states in ZnO-based materials. ESR signal at g = 2.008, attributed to oxygen-vacancy–associated trapped electrons in ZnO^95^. In the synthesized photocatalyst, vacancies enhance the absorption of visible light, facilitate charge transfer, and suppress recombination of electron–hole pairs, which increases photocatalytic efficiency across the entire solar spectrum, thus enhancing the activity of the photocatalyst^96^. The reduced ESR intensity after CuO incorporation indicates electron consumption through interfacial migration rather than defect accumulation, as shown in Fig. 4f.

**Fig. 5:**
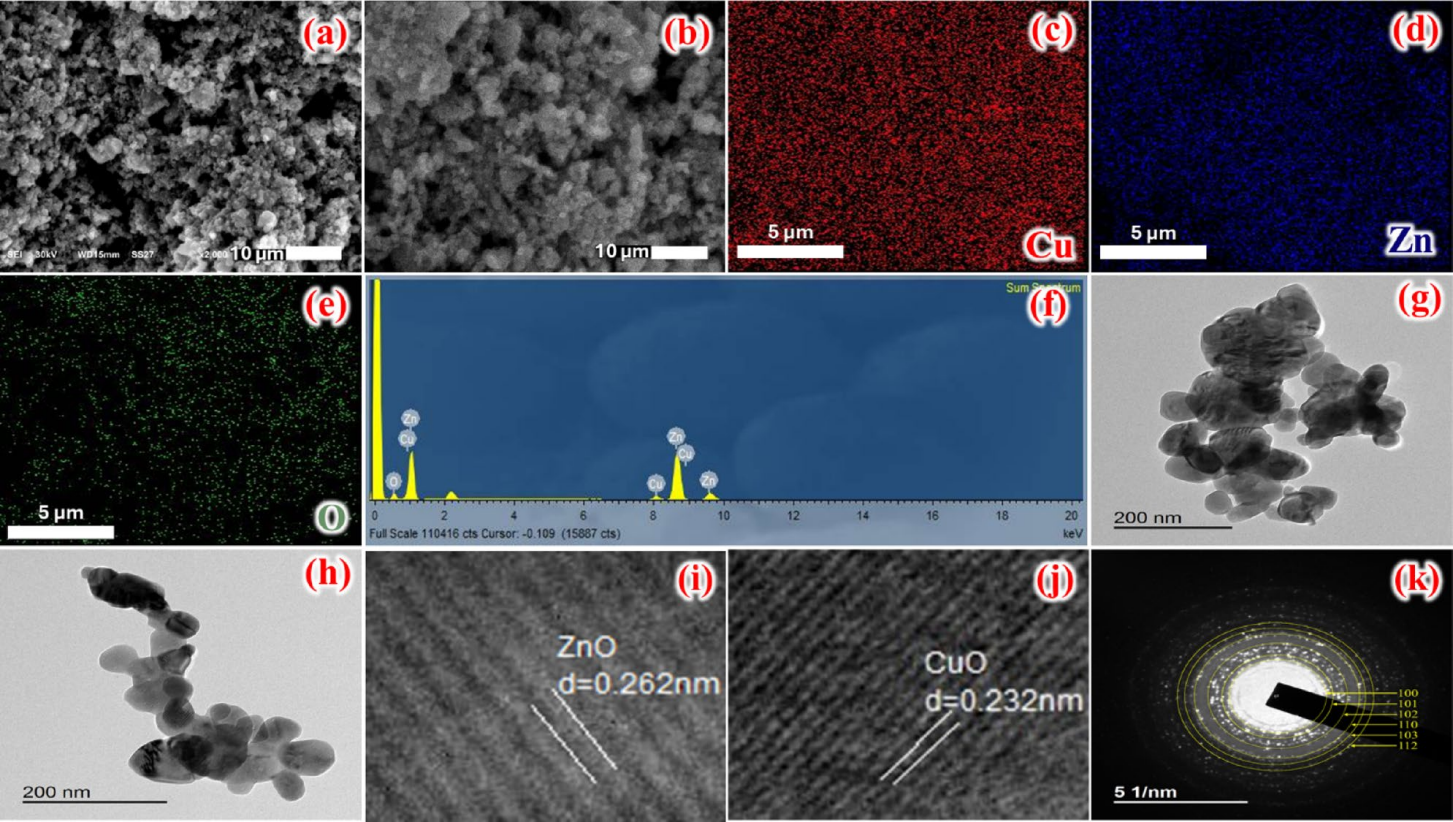
(**a** and **b**) SEM images, elemental mapping of (**c**) Cu, (**d**) Zn and (**e**) O, (**f**) EDX, (**h** and **g**) TEM images, (**i** and **j**) d-spacing of ZnO and CuO, and (k) SAED spectrum of CuO_0.25_@ZnO nanocomposite.

SEM is a powerful technique for determining a photocatalyst’s surface morphology as well as its fundamental physical properties, such as particle shape and size distribution. The fabricated oxides are obtained separately rather than by preparing a bimetallic oxide. The morphologies exhibit higher surface roughness and agglomeration, suggesting higher surface energy, as shown in Fig. 5a, b. The mapping of the CuO_0.25_@ZnO nanocomposite, as seen in Fig. 5c–e, revealed that the oxygen element is distributed throughout the entire mapping image. In contrast, the Zn element is more concentrated, and the Cu is sparser than at other locations on the ZnO surface. Also, the EDX spectrum revealed the existence of O, Cu, and Zn in the fabricated photocatalyst with atomic percentages of 48.48%, 5.28%, and 46.24%, respectively, as displayed in Fig. 5f.

TEM images provide the morphology and size characteristics of the CuO_0.25_@ZnO nanocomposite, which appears as well-separated spherical particles with uniformly distributed CuO nanoparticles on the ZnO surface, as depicted in Fig. 5g, h. This morphology increases the density of active surface sites and promotes efficient heterointerface formation. Such morphology is particularly beneficial for photocatalytic applications, as it shortens the migration distance of photogenerated charge carriers and facilitates rapid interfacial charge transfer. Furthermore, moderate particle size and controlled agglomeration behavior enhance light-absorption efficiency and improve dye-molecule adsorption on the surface, thereby increasing photocatalytic reaction kinetics. Using the histogram analysis, the particle size is calculated to be 68.5 nm, as displayed in Fig.S3. From the HRTEM image, d-space values were calculated to be 0.262 nm and 0.232 nm for ZnO (002) and CuO (111), respectively, as presented in Fig. 5i and j. Additionally, the d-spacing values were well matched to the XRD results, and the crystalline planes were well characterized by SAED and were consistent with the XRD reflections (100), (101), (102), (110), (103), and (112), as shown in Fig. 5k.

### Photocatalytic degradation measurements

**Fig. 6:**
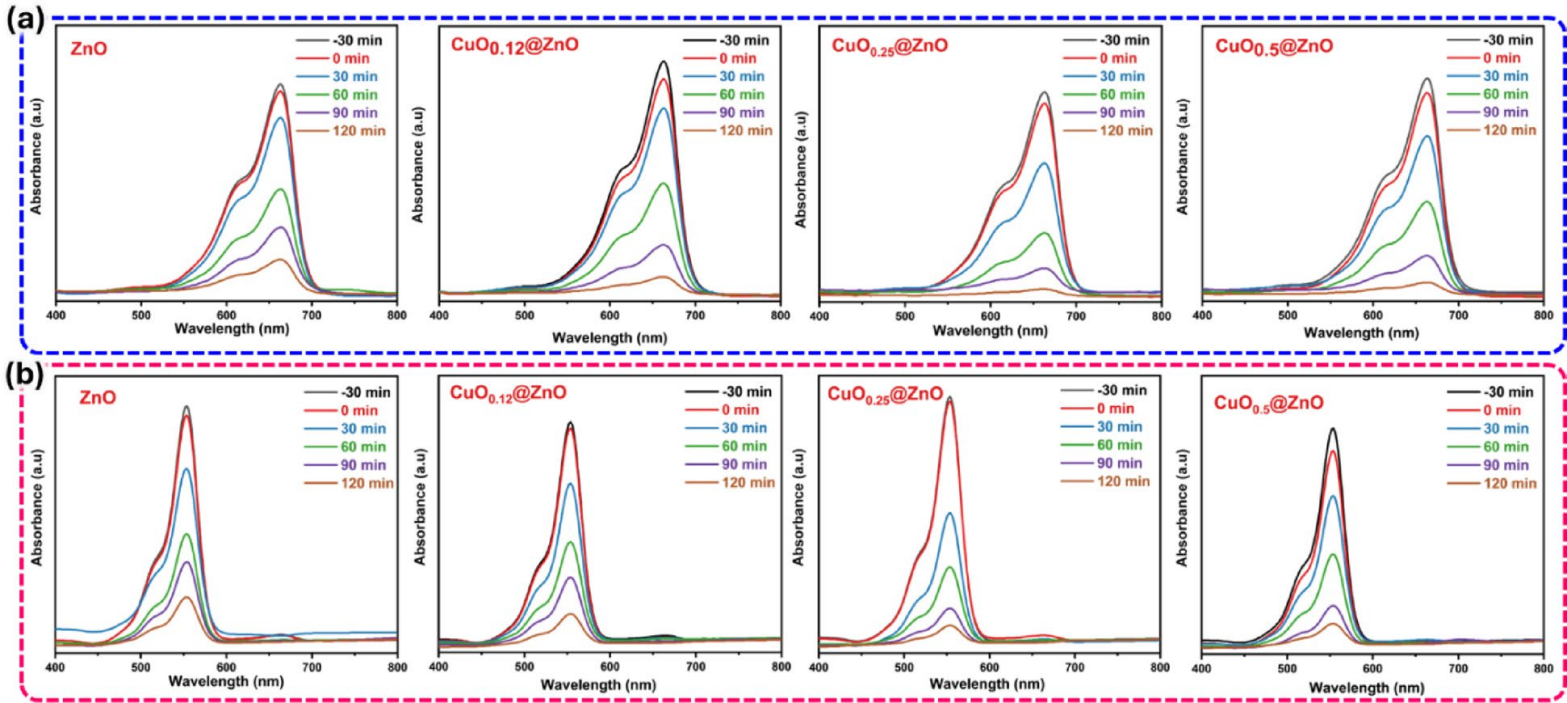
UV–visible spectra of ZnO and composites used in the degradation of (**a**) MB and (**b**) RhB dyes under the same conditions.

**Fig. 7:**
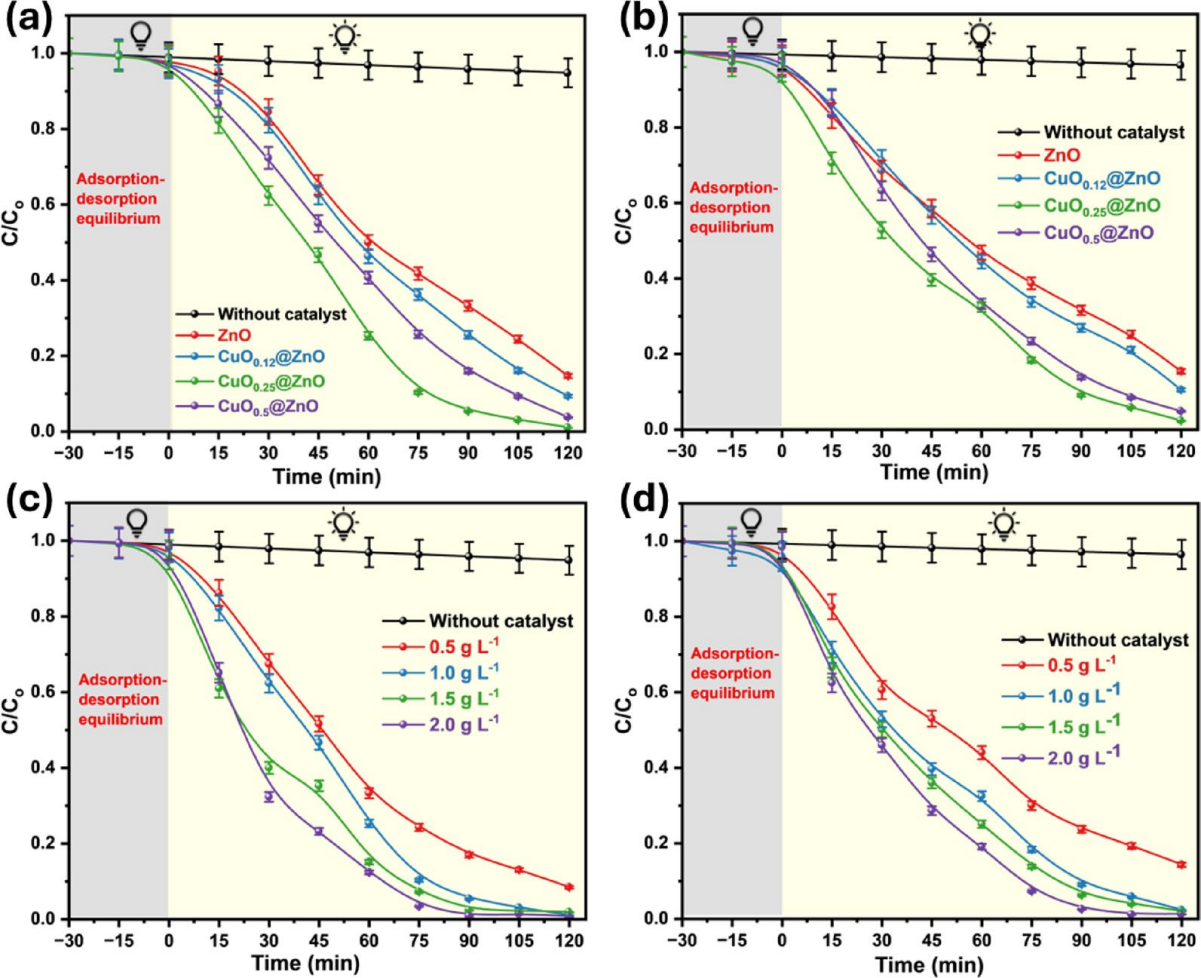
Photocatalytic efficiencies using different ratios of CuO loaded on ZnO nanoparticles used in the degradation of (**a**) MB and (**b**) RhB dyes, and the effect of photocatalyst concentration on the degradation of (**c**) MB and (**d**) RhB dyes.

The degradation performance of the nanocomposites can be estimated through the decomposition of MB and RhB dyes (10 mg L^−1^) with a catalyst dose of 1.0 g L^−1^ at an initial pH of 7. The photocatalytic degradation rates for pure ZnO nanoparticles reached nearly 85.3% and 84.5% for MB and RhB, respectively, after 120 min of irradiation. These degradation efficiencies increased with the use of CuO nanoparticles as a dopant in varying percentages. It was found that the concentration of 25.0 wt% CuO in the synthesized CuO_0.25_@ZnO nanocomposites exhibited higher degradation rates of about 99% and 97.7% from MB and RhB dyes, respectively, compared to other ratios of nanocomposites (Fig. 6a, b). However, the photocatalytic degradation efficiency decreased with increasing CuO nanoparticle content in the as-prepared composites. High CuO loading leads to particle aggregation and coverage of the ZnO active surface, which reduces the number of accessible active sites. In addition, excessive CuO can partially shield ZnO from light irradiation and block active surface sites, thereby limiting charge generation and pollutant adsorption. Moreover, high CuO content increases the photogenerated charge-carrier recombination, thereby preventing electron and hole migration to the material surface and decreasing photocatalytic efficiency, as displayed in Fig. 7a, b^97^. Photolysis essays are crucial for distinguishing the effects of direct light irradiation from the material’s photocatalytic activity. The photolysis experiments were performed under identical conditions in the absence of a photocatalyst, and the data indicate that the target pollutant undergoes little or no photodegradation, suggesting that photocatalytic processes are the primary source of the observed removal efficiency when the catalyst is present.

Photocatalyst concentration is one of the critical parameters that influence the degradation kinetics. Four distinct concentrations of CuO_0.25_@ZnO photocatalyst of 0.5, 1.0, 1.5, and 2.0 g L^−1^, as well as fixed concentrations of organic dyes (10 mg L^−1^), were used in the experiment to study the effect of catalyst dose. Figure 7c and d show that increasing the photocatalyst concentration from 0.5 to 2.0 g L^−1^ improves the degradation efficiencies of organic dyes, as evidenced by the UV–visible spectra in Fig. S5 and S6. High catalyst concentrations may increase photon adsorption on active sites and the adhesion of dye molecules to the photocatalyst surface^98–101^.

**Fig. 8:**
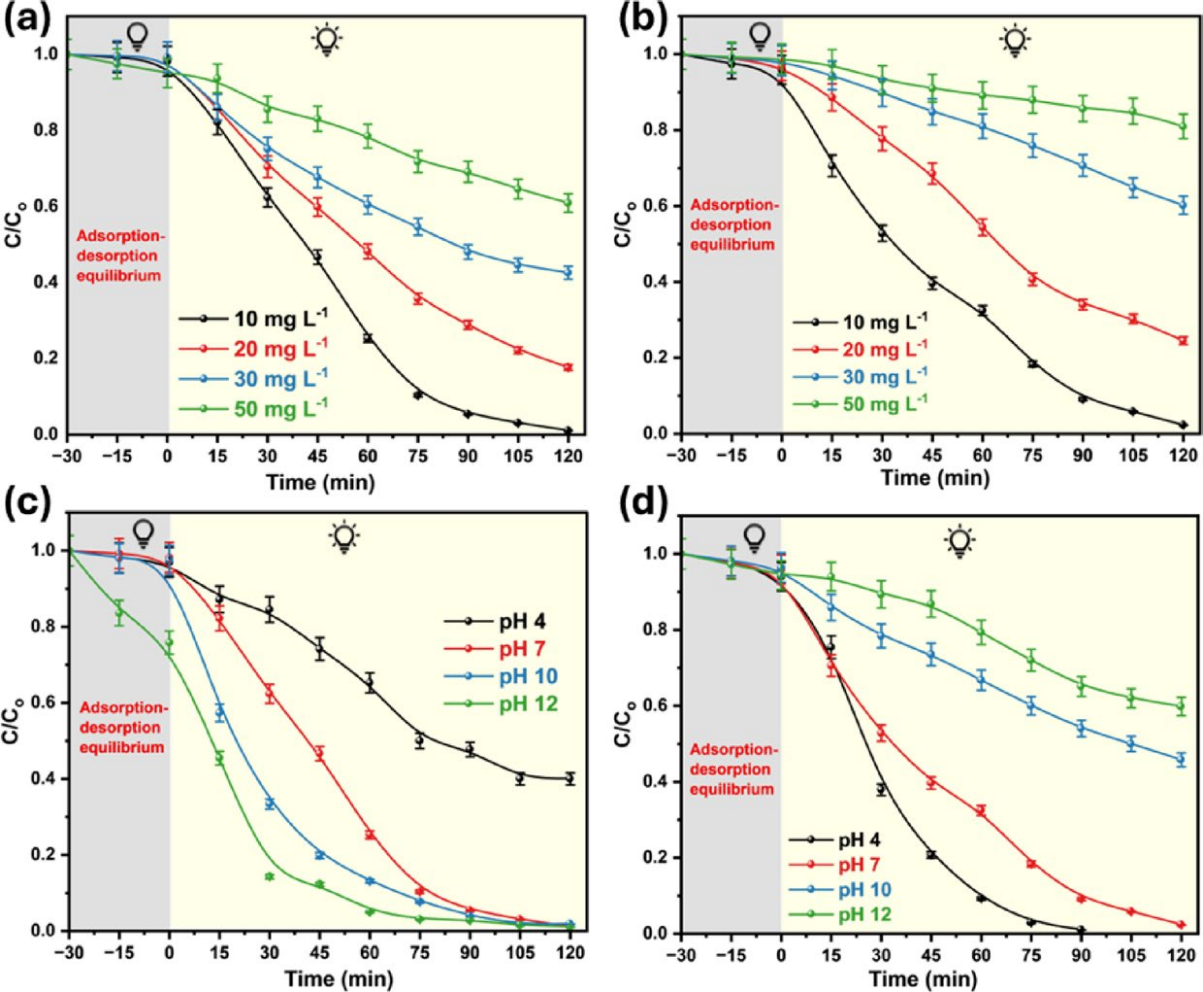
Photocatalytic degradation at different concentrations of (**a**) MB and (**b**) RhB dyes and effect of pH change on degradation of (**c**) MB and (**d**) RhB dyes using CuO_0.25_@ZnO.

To investigate the degradation efficiency, two series of MB and RhB dye solutions at concentrations of 10, 20, 30, and 50 mg L^−1^ were studied under constant dosage, temperature, and pH, as shown in Fig. 8a and b. Raising the initial dye concentration reduces the degradation efficiency, as seen in Fig. S7 and S8. This could be because less incident light is available on the photocatalyst surface at higher color intensities. Furthermore, dye molecules blocking the catalyst’s active sites further reduce its efficiency, slowing the reaction rate and leaving considerable amounts of undegraded dye^102^.

Photocatalytic activity is greatly influenced by the pH value of the dye solution. They are essential for altering the surface carriers of the photocatalyst and influencing interactions with pollutants and hazardous materials. These experiments are performed at pH 4, 7, 10, and 12 using diluted HCl and NaOH solutions. The photocatalytic decomposition of MB dye exhibited a low degradation rate in an acidic medium, but increased at higher pH, reaching a maximum at pH 12. The addition of HO^−^ ions allowed about 97.3% of the MB dye to degrade after 90 min, as illustrated in Fig. 8c. This accelerated dye degradation due to the strong electrostatic contact between the negatively charged photocatalyst surface and the cationic dye^102,103^. On the contrary, the degradation percentages of RhB dye were recorded to be 99% at pH 4 after 90 min, as illustrated in Fig. 8d. This could be because the catalyst’s surface is positively charged in an acidic medium. At the same time, the majority of RhB’s carboxyl groups ionize to generate negatively charged species^104^. Therefore, the optimal pH values for the degradation of MB and RhB dyes were 12 and 4, as shown in Fig. S9 and S10, respectively.

**Fig. 9:**
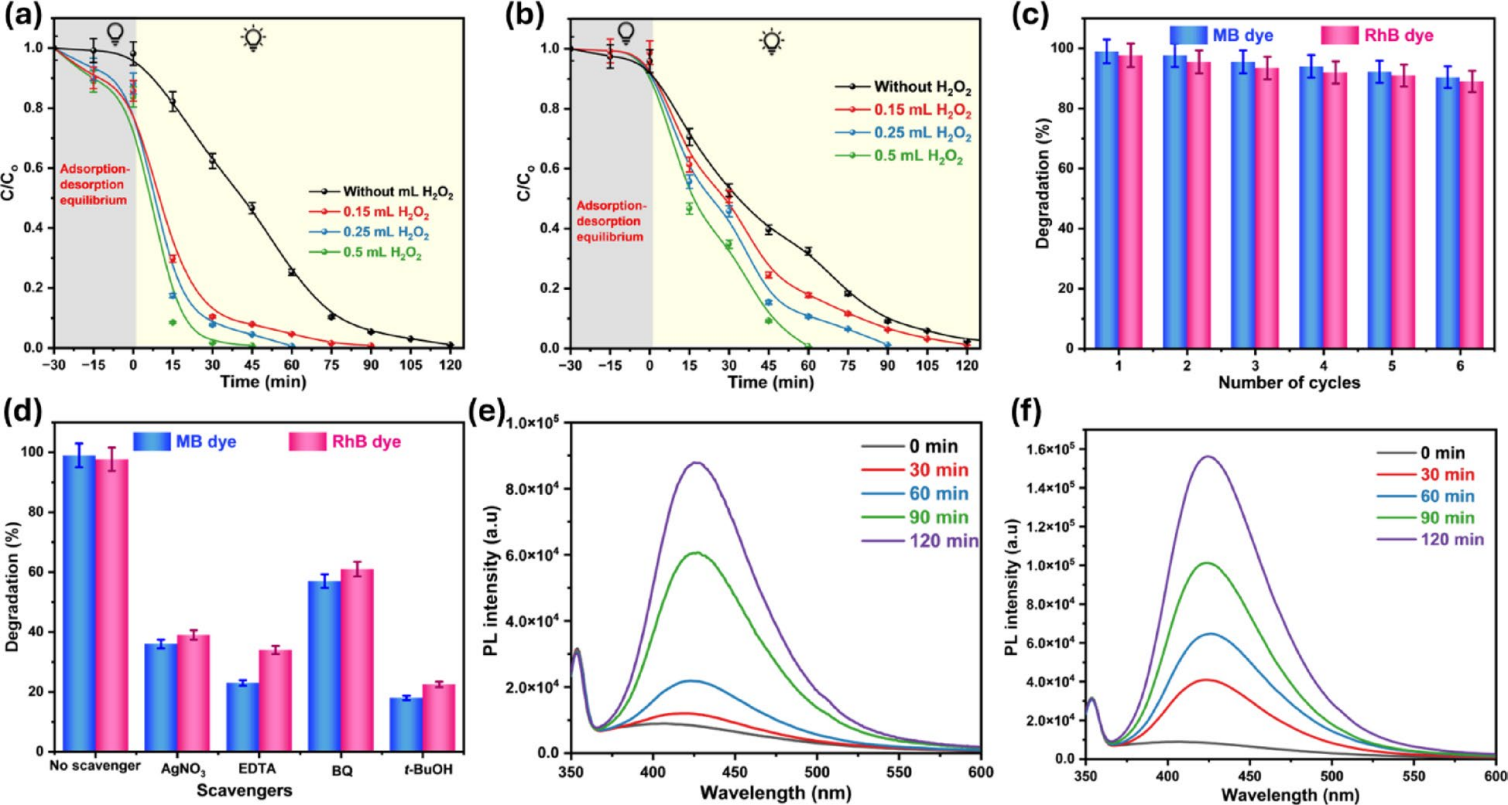
Effect of H_2_O_2_ concentrations on the photocatalytic degradation of (**a**) MB and (**b**) RhB dyes using CuO_0.25_@ZnO nanocomposite, (**c**) reusability of CuO_0.25_@ZnO nanocomposite for photodegradation of MB and RhB dyes over six continuous cycles, and (**d**) photocatalytic performance in the presence of different scavengers, PL spectra of terephthalic acid for (**e**) ZnO, and (f) CuO_0.25_@ZnO.

One of the most widely employed electron acceptors to enhance the photocatalytic process is hydrogen peroxide, which promotes the generation of hydroxyl radicals (HO^•^) during the photochemical reaction^105,106^. As a result, the process will be more efficient, and electron–hole recombination will decrease. Under optimal conditions, three distinct H_2_O_2_ concentrations were introduced to the photocatalytic reactor to examine their effects on the decomposition of organic dyes. The results show that the addition of H_2_O_2_ to the reaction mixture improves efficiency and shortens the dye-degradation time, as displayed in Fig. 9a, b. After 30 min, approximately 91.5%, 92.25%, and 98.36% of MB dye were degraded using 0.15, 0.25, and 0.5 mL H_2_O_2_, and about 82.23%, 89.38%, and 99.45% of RhB dye, respectively, after 60 min, as displayed in Fig. S11 and S12. H_2_O_2_ molecules serve two essential functions in dye degradation. First, it promotes the production of HO^•^, which prevents photo-induced electron and hole recombination as illustrated in Eq. (4).

4 $$e^{ - } \left( {CuO@ZnO } \right) + H_{2} O_{2} \to CuO@ZnO + HO^{ - } + HO^{ \cdot }$$

Secondly, H_2_O_2_ promotes charge separation by accepting electrons in the CB, forming HO^•^, as explained in Eqs. (5 and 6)^102,107^.

5 $$H_{2} O_{2} + e^{ - } \to HO^{ - } + HO^{ \cdot }$$

6 $$H_{2} O_{2} + O_{2}^{ \cdot - } \to HO^{ - } + HO^{ \cdot } + O_{2}$$

A scavenger method was used to detect the reactive species responsible for organic dye degradation^83^. The photocatalytic process involves four primary active species: e^−^, h^+^, $\left( {{\mathrm{O}}_{2}^{ \bullet - } } \right)$, and HO^•^. The photodegradation rates reduced to 39% and 36%, respectively, after AgNO_3_ was added to the RhB and MB dye solutions. This is because AgNO_3_ functions as an electron scavenger, trapping electrons and preventing the production of ${\mathrm{O}}_{2}^{ \bullet - }$. Additionally, EDTA-2Na performs as a hole scavenger, preventing MB and RhB dye degradation by 23% and 34%, respectively. The HO^•^ radicals can be produced through the interaction of holes with ${\mathrm{H}\mathrm{O}}^{-}$ on the CuO_0.25_@ZnO surface or with water molecules. Consequently, the inclusion of tert-butyl alcohol (*t*-BuOH) in the reaction system, which captures photogenerated HO^•^ species, significantly diminished the degradation of RhB and MB dyes to 22.5% and 18%, respectively. Additionally, the photocatalytic degradation rates of RhB and MB dyes were reduced to 61% and 57%, respectively, in the case of BQ, which acts as a ${\mathrm{O}}_{2}^{ \bullet - }$ scavenger by the following straightforward electron transfer mechanism^108^:

7 $$BQ + O_{2}^{ \cdot - } \to BQ^{ \cdot - } + O_{2}$$

The addition of scavengers decreased degradation efficiency in all instances, indicating that all species (e^−^, h^+^, HO^•^, and ${\mathrm{O}}_{2}^{ \bullet - }$) are involved in the degradation process, with HO^•^ being the primary reactive molecule accountable for the deterioration of the organic dyes by the CuO_0.25_@ZnO nanocomposite, as illustrated in Fig. 9d^109^.

The dominant role of HO^•^ radicals observed in scavenger and photocatalytic experiments is evaluated quantitatively using probe molecules such as terephthalic acid. The fluorescence intensity of 2-hydroxyterephthalic (HTA) at 425 nm rose with increasing irradiation time, suggesting an increase in HTA concentration. Generally, the peak intensity of HTA was proportional to the amount of HO^•^ radicals produced. Therefore, by prolonging the irradiation time, the HO^•^ radical concentration increased^110^. As shown in Fig. 9e and f, after 120 min irradiation, the HTA peak intensity in the CuO_0.25_@ZnO composite was higher than that in the pure ZnO. These results confirmed that the oxidation of H_2_O by the photogenerated holes on the CuO_0.25_@ZnO surface produces more HO^•^ radicals, which match the scavenger experiment.

Reusable photocatalysts are necessary for different economic industrial applications. The reusability of the CuO_0.25_@ZnO nanocomposite was examined over six cycles. To perform the next cycle, the composite was centrifuged, washed, and dried after every photocatalytic cycle. The photocatalyst was reintroduced to the dye solutions under optimized conditions. In the second reuse of the photocatalyst, efficiency decreased to 97.7% and 95.5% for MB and RhB, respectively. In the six-cycle, the photocatalyst maintained high removal efficiency, supporting its reusability, as shown in Fig. 9c and S13. As a result, the generated photocatalysts are highly effective and can be reused repeatedly to degrade various organic dyes. Additionally, XRD analysis was performed on the reused catalyst after six cycles, as shown in Fig. S4, confirming that the crystal structure remains unchanged after repeated use. This provides strong evidence of the material’s inherent stability. These findings suggest that the CuO_0.25_@ZnO heterostructure is sufficiently robust to support future long-term and continuous-flow studies.

### Kinetic studies

**Fig. 10:**
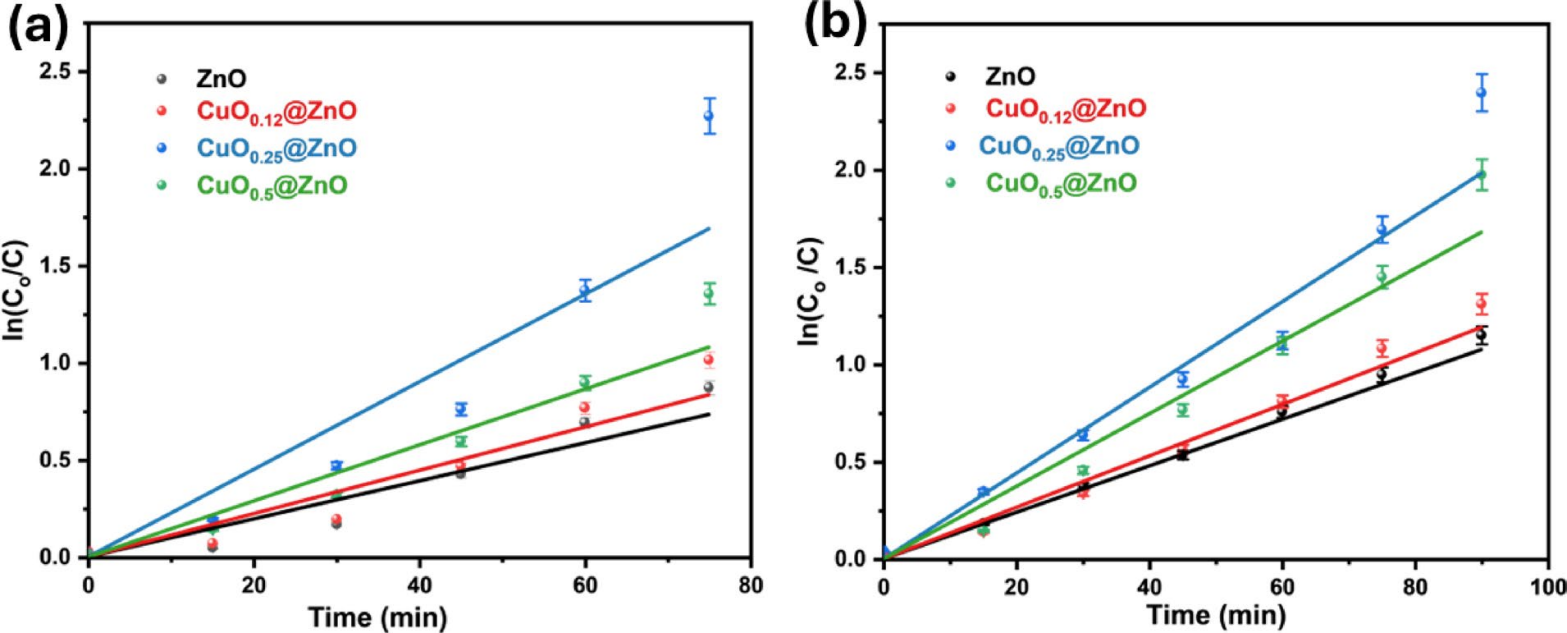
First-order kinetic model for differently prepared nanocomposites for degradation of (**a**) MB and (**b**) RhB dyes.

Moreover, we applied pseudo-first-order models based on the Langmuir–Hinshelwood (L–H) mechanism at low pollutant concentrations. Also, pseudo-second-order models to investigate the photocatalytic degradation kinetics of organic dyes, as expressed in Eqs. (8 and 9) as follows:

8 $$\ln \frac{{{\mathrm{C}}_{{\mathrm{o}}} }}{{{\mathrm{C}}_{{\mathrm{t}}} }} = {\mathrm{kt}}$$

9 $$\frac{1}{{C_{t} }} - \frac{1}{{C_{o } }} = kt$$

where; C_o_ and C_t_ denote the initial concentration and concentration at time t, respectively, and k signifies the degradation rate constant (min^−1^). The linearization of the results confirms pseudo-first-order kinetics for the photodegradation of MB and RhB. Good correlation to the first pseudo-order kinetics with values of 0.9477 and 0.984 for decomposition of MB and RhB dyes, respectively (Fig. 10 a and b). Furthermore, CuO_0.25_@ZnO has a k value of approximately 0.025 and 0.024 min^−1^ in the case of MB and RhB dyes, respectively, higher than those of pure ZnO (0.01067 and 0.0128 min^−1^). The results of this study demonstrate that CuO increases conductivity and photoexcited electron–hole separation to modulate the photocatalytic features of the CuO_0.25_@ZnO. But second-order models show lower correlation than the first pseudo-order kinetics, with values of 0.5967 and 0.6823 for the decomposition of MB and RhB dyes, respectively (Fig. S14a and b). Also, the pseudo-first-order kinetic model for CuO_0.25_@ZnO nanocomposite at 6 successive cycles for the degradation of MB and RhB dyes is presented in Fig. S15a and b.

### Photoelectrochemical measurements

**Fig. 11:**
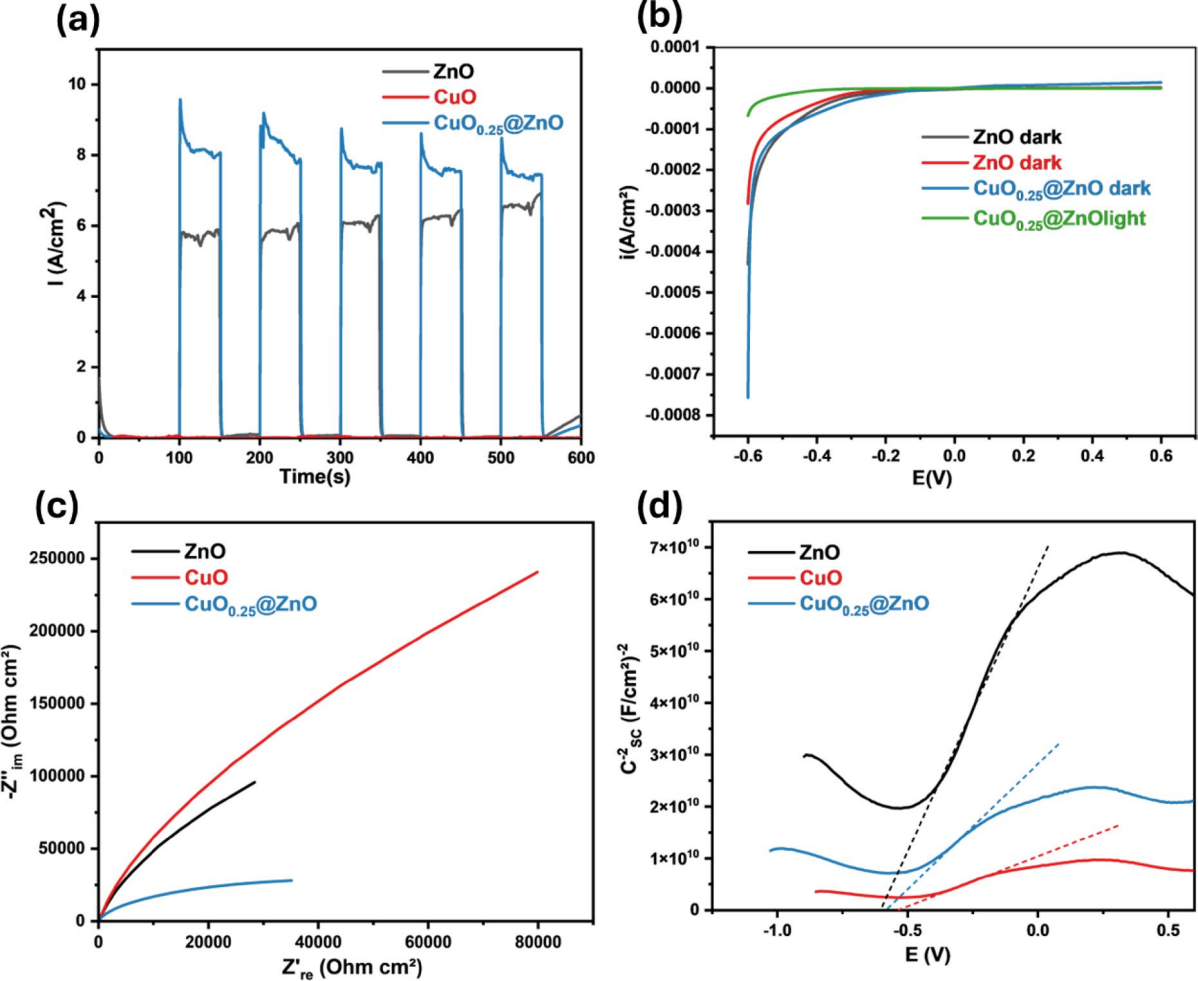
Photoelectrochemical studies of prepared photocatalysts (**a**) I-t, (**b**) LSV, (**c**) EIS, and (**d**) Mott-Schottky plots.

The charge-carrier transport in the generated photocatalysts was investigated using photocurrent response experiments. (I-t) plots for ZnO, CuO, and CuO_0.25_@ZnO at a bias potential of 0 V vs Ag/AgCl are displayed in Fig. 11a after many on/off cycles of intermittent illumination using a Xe lamp. It was observed that the current increased when the photocatalyst electrode was exposed to light, because light excited a large number of electrons and holes in the photocatalyst^111^. The photocurrent response of CuO_0.25_@ZnO photocatalyst was about 9.5 $\times$ 10^−7^ A cm^−2^ higher than pure ZnO of 5.7 $\times$ 10^–7^ A cm^−2^, indicating that CuO_0.25_@ZnO nanocomposite extends the lifespan of photogenerated charge carriers, which enhances generation, separation, and migration of photogenerated charge carriers under illumination in comparison to ZnO. However, the CuO electrode shows a low photocurrent response due to its narrow band gap, low charge carrier mobility, short lifetime of photoexcited charge carriers, and poor photostability, which significantly limit its performance^112–114^.

Moreover, LSV was performed on ZnO and CuO_0.25_@ZnO nanocomposite in a 0.5 M Na_2_SO_4_ electrolyte solution under dark and light conditions. As displayed in Fig. 11b, all samples exhibit low current density under dark conditions. The absorption of visible light enhances the photocurrent density of the CuO_0.25_@ZnO nanocomposite, indicating that CuO can generate and transfer photoinduced electrons and holes from the VB to the CB under irradiation^115^. This suggests that CuO_0.25_@ZnO has higher photocatalytic performance than pure ZnO.

Additionally, the kinetics of electron transfer at the photoanode-electrolyte interface have been investigated using EIS. Effective charge transfer is indicated by a shorter radius in the semicircles used to measure electron-transfer resistance^116^. As noticed in Fig. 11c, CuO_0.25_@ZnO has a smaller arc radius on Nyquist plots in the following sequence: CuO > ZnO > CuO_0.25_@ZnO photocatalysts, which indicates that the interfacial charge migration ability of CuO_0.25_@ZnO was faster than that of ZnO. The doped CuO nanoparticles improved charge-carrier separation, confined electrons, and impeded electron–hole pair recombination, as evidenced by the PL spectra^117^.

The semiconducting characteristics of the photocatalysts were examined using the MS representation, which relates potential to the semiconductor-electrolyte interface, as described by Eq. (10).

10 $$C_{SC}^{ - 2} = \left( {\frac{2}{{\varepsilon \varepsilon_{^\circ } qN_{D} }}} \right)\left( {V - V_{fb} - \frac{KT}{e}} \right)$$

where; N_D_ stands for electron carrier density (cm^−3^), ε for dielectric constant, C_SC_ for space charge capacitance (F cm^−2^), εₒ are relative and vacuum permittivity (8.85 $\times$ 10^−14^ F cm^−1^), respectively, e is the electron charge (1.602 $\times$ 10^−19^ C), T is the absolute temperature (K), and k is the Boltzmann constant (1.38 $\times$ 10^−23^ J K^−1^), V and V_fb_ are the applied and flat band potentials, respectively^118,119^. MS analysis is a practical electrochemical method for identifying the type of semiconductor, flat-bandgap, and donor concentration, all of which are essential for improving the photocatalytic processes^120^. The flat-band potential was determined by intersecting the tangent to the MS plot with the x-axis. As seen in Fig. 11d, the flat band potential of the CuO_0.25_@ZnO nanocomposite was found to shift toward the more positive side in comparison to that of pure ZnO. This change in the Fermi level toward the CB improves the efficiency of charge transfer by increasing the mobility of charge carriers at the electrode/electrolyte interface and decreasing electron/hole pair recombination^121,122^. Furthermore, because the CB of CuO is more positive than that of ZnO nanoparticles, it exhibited a higher degradation potential^123^. The positive slopes of CuO, ZnO, and CuO_0.25_@ZnO were obtained by fitting the linear portion of the MS curves, providing qualitative evidence that the prepared semiconductors are n-type and the heterojunction is n–n type^124^. Table 1 lists the slope, N_D_, and V_fb_ values derived from the MS plots. The incorporation of CuO decreased the slope of CuO_0.25_@ZnO nanocomposite, suggesting a higher donor density. The donor density increased from 1.45 $\times$ 10^19^ cm^−3^ for ZnO to 3.98 $\times$ 10^19^ cm^−3^ for the CuO_0.25_@ZnO composite, corresponding to an approximately 2.7-fold increase in carrier concentration. This increase in donor density indicates greater availability of free charge carriers, thereby enhancing electrical conductivity and facilitating faster interfacial charge transport, which subsequently improves the photocatalytic performance of CuO_0.25_@ZnO photocatalyst. According to Eq. (11), the space-charge layer width (W_SCL_) is calculated using the Poisson equation based on N_D_ and V_fb_ factors.

11 $$W_{SCL} = \sqrt {\frac{{2\varepsilon \varepsilon_{^\circ } \left( {V - V_{fb} } \right)}}{{eN_{D} }}}$$

**Table 1:** The values of ND, Vfb, and WSCL were obtained from Mott–Schottky plots.

Sample	Slope	N_D_ (cm^−3^)	V_fb_ (V)	W_SCL_ (μm)
CuO	1.74 × 10^10^	9.55 × 10^19^	− 0.50	0.982
ZnO	1.15 × 10^11^	1.45 × 10^19^	− 0.60	2.76
CuO_0.25_@ZnO	4.17 × 10^10^	3.98 × 10^19^	− 0.57	1.63

According to Table 1, the WSCL value of CuO_0.25_@ZnO was reduced by 59% compared to pure ZnO, which augmented charge carrier transfer and boosted the photoelectrochemical activity of the synthesized photoanodes^120^.

### Photocatalytic degradation mechanism

The photocatalytic degradation process is generally explained in terms of two electron–hole migration mechanisms: type-II and Z-scheme electron–hole transfers. ZnO and CuO absorb the incident photons, which generate h^+^ and e^−^ in VB and CB, respectively. Based on the calculated band edge positions (CB and VB) from MS curves, combined with optical band gap values. Fermi energy levels (E_f_) in n-type semiconductors are very close to the CB^125,126^. Thus, the CBs of ZnO and CuO were positioned at − 0.6 and − 0.5 (V vs NHE), respectively. The VB of ZnO and CuO were calculated to be + 2.5 and + 0.6 (V vs NHE), respectively, using the following Eq. (12):

12 $$E_{VB } = E_{CB } + E_{g}$$

**Fig. 12:**
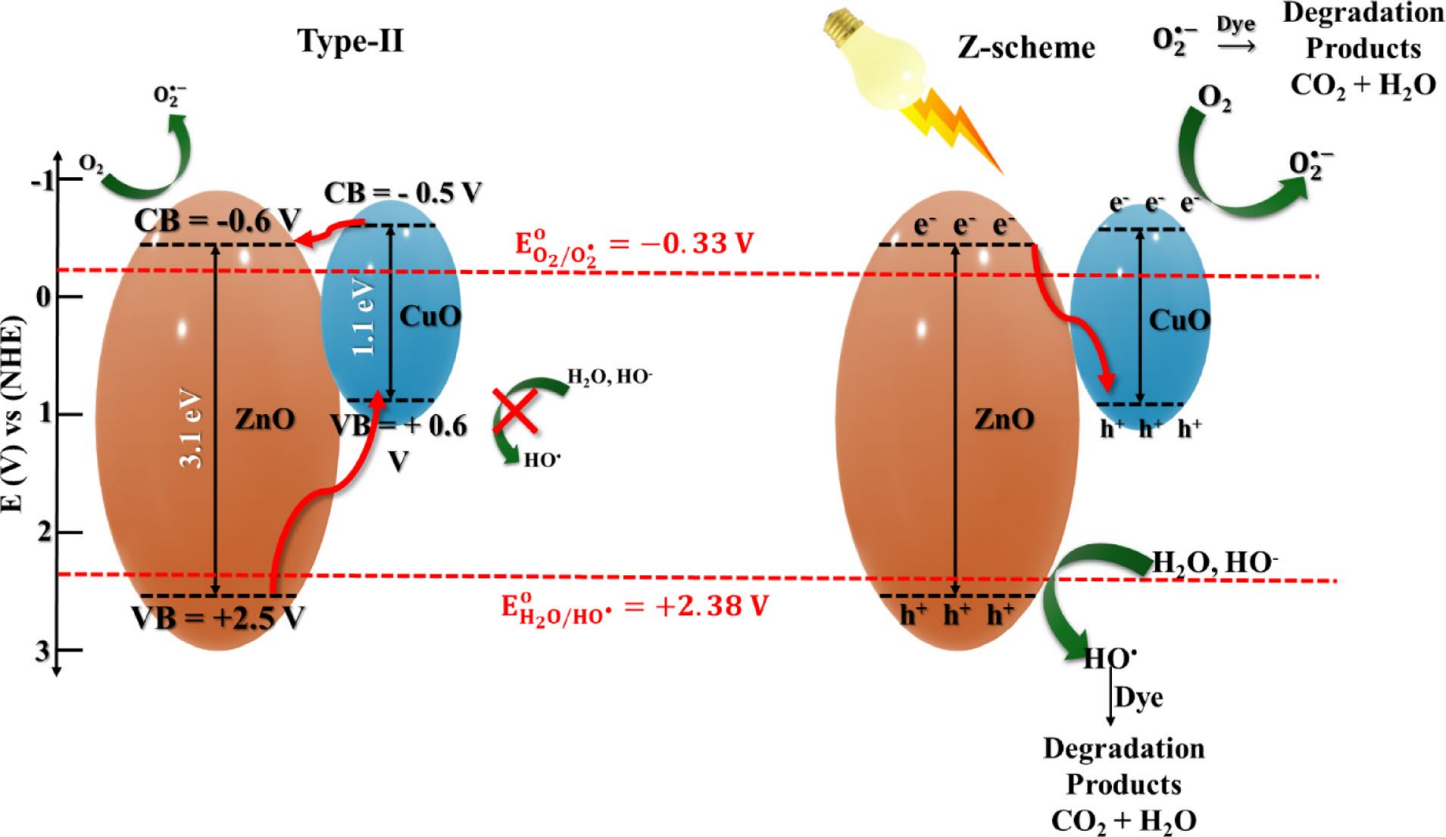
Schematic representation of CuO_0.25_@ZnO charge transfer under solar light illumination.

Therefore, a Z-scheme heterojunction, compared to a traditional type-II heterojunction, more accurately describes the charge-transfer mechanism. The Z-scheme heterojunction is explained by the recombination of ${\mathrm{e}}^{-}$ in the CB of ZnO with h^+^ in the VB of CuO by the electrostatic attraction. In this way, charge carriers are effectively separated across individual materials, thereby extending their lifetime. The VB potential of ZnO (+ 2.5 V vs NHE) is more positive than ($E_{{H_{2} O/HO^{ \cdot } }}^{^\circ }$= + 2.38 V), which enables photogenerated h⁺ to oxidize HO⁻, leading to the formation of HO^•^. The more negative CB potential of CuO (− 0.5 V vs NHE), relative to ($E_{{O_{2} /O_{2}^{ \bullet - } }}^{^\circ }$= − 0.33 V), facilitates the reduction of O_2_ to ${\mathrm{O}}_{2}^{ \bullet - }$, as displayed in Fig. 12. This mechanism preserves strong redox ability and explains the dominant formation of hydroxyl radicals observed experimentally (Table 2).

**Table 2:** Comparison between the prepared photocatalyst and others in the literature:.

Photocatalyst	Catalyst dose (mg)	Dye (Concentration)	Time of degradation	Rate constant (k)	Efficiency [%]	Ref
CuO-ZnO (CZ-NC)	50 mg	RhB (10 ppm)	105 min	0.00048 s^−1^	96.1%	^78^
CuO − ZnO	20 mg	MB (20 ppm)	105 min	0.017 min^−1^	82.0%	^73^
β-CD-CuO/ZnO	50 mg	MB MG (1 × 10 ^−5^ M)	180 min	0.0122 min^−1^	89.15% 79.90%	^83^
Cu_2_O/ZnO	100 mg	MO	240 min	0.022 min^−1^	98.0%	^127^
CuO/ZnO (4:6)	10 mg	MB (5 ppm)	150 min	0.0295 min^−1^	93.0%	^89^
CZ-30	20 mg	MB (25 ppm)	80 min	0.0529 min^−1^	98.6%	^128^
CuO–ZnO	25 mg	MB 10^–3^ M	120 min	0.0235 min^−1^	95.6	^129^
ZnO/CuO	40 mg	MB 20 ppm	60 min	0.0223 min^−1^	93.0%	^130^
CuO–ZnO	50 mg	MB 15 ppm	120 min	0.01948 min^−1^	93.66%	^131^
Cu_2_O/ZnO	20 mg	MB 10 ppm	60 min	0.022 min^−1^	73.3%	^132^
CuO_0.25_@ZnO	50 mg	MB 10 ppm	120 min	0.025 min^−1^	99%	Current work
		RhB 10 ppm		0.024 min^−1^	97.7%	

In a type-II heterojunction, ${\mathrm{e}}^{ - }$ migrate from the CB of CuO to that of ZnO, while h^+^ migrates from the VB of ZnO to CuO. However, the holes accumulated in the VB of CuO cannot oxidize H₂O to generate HO^•^ radicals because the VB potential of CuO (+ 0.6 V vs NHE) is significantly lower than ($E_{{H_{2} O/HO^{ \bullet } }}^{^\circ }$= + 2.38 V). So, the hole-oxidation capability of the CuO_0.25_@ZnO photocatalyst decreased. This result does not align with those from the scavenger experiments and the fluorescent intensity of HTA. Thus, type II electron transfer cannot explain the photocatalytic mechanism of the CuO_0.25_@ZnO photocatalyst^76,77,110^. The generated active radicals break down the organic pollutants of dyes on the surface of the CuO_0.25_@ZnO photocatalyst, converting them into harmless molecules such as H_2_O and CO_2_, as illustrated in Eqs. (13–17).

13 $$CuO@ZnO + h\upsilon \to CuO@ZnO^{*} + h_{VB}^{ + } + e_{CB}^{ - }$$

14 $${\mathrm{H}}_{2} {\mathrm{O}} \rightleftharpoons {\mathrm{H}}^{ + } + {\mathrm{HO}}^{ - }$$

15 $$HO^{ - } + ZnO\left( {h_{VB}^{ + } } \right) \to HO^{ \cdot }$$

16 $$O_{2} + CuO + \left( {e_{CB}^{ - } } \right) \to O_{2}^{ \cdot - }$$

17 $$HO^{ \cdot } + O_{2}^{ \cdot - } + \, Organic \, pollu\tan ts \, \to \, H_{2} O \, +$$

## Conclusion

A straightforward technique was employed to synthesize CuO@ZnO n–n heterojunction-based Cu-BDC frameworks derived from waste via solvothermal methods. Different quantities of CuO nanoparticles were integrated into ZnO to improve photocatalytic degradation efficiency. Moreover, CuO_0.25_@ZnO nanocomposite exhibited good visible light absorption by lowering the band gap energy from 3.1 eV to 2.89 eV compared to other fabricated photocatalysts, thereby enhancing the decomposition rates of MB and RhB dyes to 99% and 97.7%, respectively. The photocatalytic performance of CuO_0.25_@ZnO nanocomposite was investigated under varying pH, photocatalyst dose, dye, and H_2_O_2_ concentrations. The optimal pH for dye degradation was 4 for RhB and 12 for MB. After six cycles, the structure of the fabricated photocatalyst retains its crystallinity, and the photocatalytic performance remains unchanged, illustrating its stability and recyclability. The PL and the EIS spectra indicate that CuO_0.25_@ZnO has a higher electron–hole separation and lower charge transfer resistance than pure ZnO. The photocatalytic degradation mechanism of CuO_0.25_@ZnO, described as the Z-scheme, which preserves strong redox ability and explains the dominant role of hydroxyl radicals observed in trapping experiments and in the fluorescent spectrum of HTA. This result was a guide for further preparation and development of other n-type CuO photocatalysts. In the future, the researchers are moving towards green synthesis to create high-value products from inexpensive, recyclable, renewable resources, thereby fulfilling the waste-to-value-added product principle across different applications and encouraging community waste recycling.

## Supplementary Information

Below is the link to the electronic supplementary material.

Supplementary Material 1

## Data Availability

All data generated or analyzed during this study are included in this published article (and its Supplementary Information files).
