# Supplementary material for: Valorization of domestic wastes into Cu-MOF-derived CuO@ZnO nanocomposites for sustainable photocatalytic degradation of methylene blue and rhodamine B dyes
Source: Sci Rep. 2026 May 13;16:15042. doi: 10.1038/s41598-026-51864-6 (PMC13172463; doi:10.1038/s41598-026-51864-6)
Supplement: Supplementary file 1 — Supplementary Material 1 [file 41598_2026_51864_MOESM1_ESM.docx]

**Valorization of domestic wastes into Cu-MOF-derived CuO@ZnO nanocomposites for sustainable photocatalytic degradation of methylene blue and rhodamine B dyes**

M.S. Samy^a^, H.M. Abou El Nadar^a^, E.A. Gomaa^a^, Amr Awad Ibrahim^a, b^, Mina Shawky Adly^a, b^*

*^a^Department of Chemistry, Faculty of Science, Mansoura University, Al-Mansoura 35516, Egypt.*

*^b^Energy & Desalination Center, Faculty of Science, Mansoura University, Egypt*

*E-mail:* [*mina_shawky-90@mans.edu.eg*](mailto:mina_shawky-90@mans.edu.eg)*; Tel: +20-1019197625*

Table. S1. Particle size of prepared nanocomposites using the Scherrer equation.

| **Photocatalyst** | **2θ** | **D (nm)** |
| --- | --- | --- |
| CuO | 38.761° | 35.2 |
| ZnO | 36.276° | 56.3 |
| CuO_0.12_@ZnO | 36.298° | 49.5 |
| CuO_0.25_@ZnO | 36.310° | 61.4 |
| CuO_0.5_@ZnO | 36.319° | 59.4 |


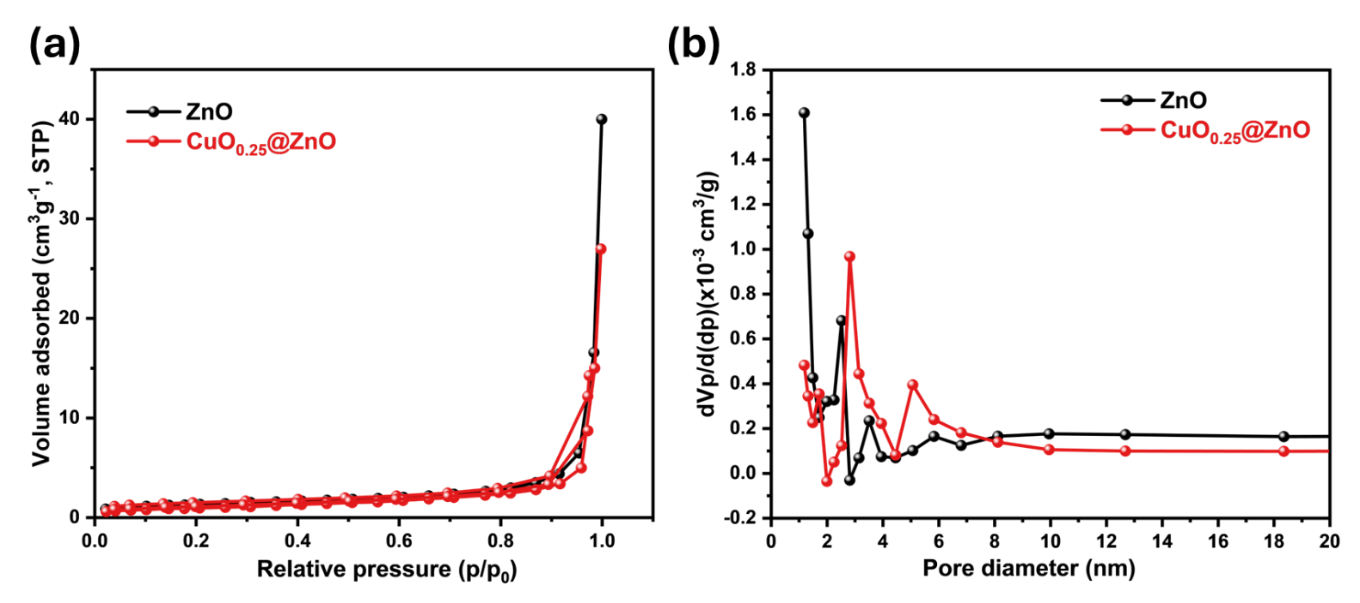


Fig. S1. (a) N_2_ adsorption–desorption isotherm and (b) Barrett-Joiner-Halenda (BJH) pore size distribution plots of ZnO and CuO_0.25_@ZnO nanocomposite.

Fig. S2. UV-vis diffuse absorbance spectra of CuO, ZnO NPs, and nanocomposites.

Fig. S3. The particle size distribution of the nanocomposite.


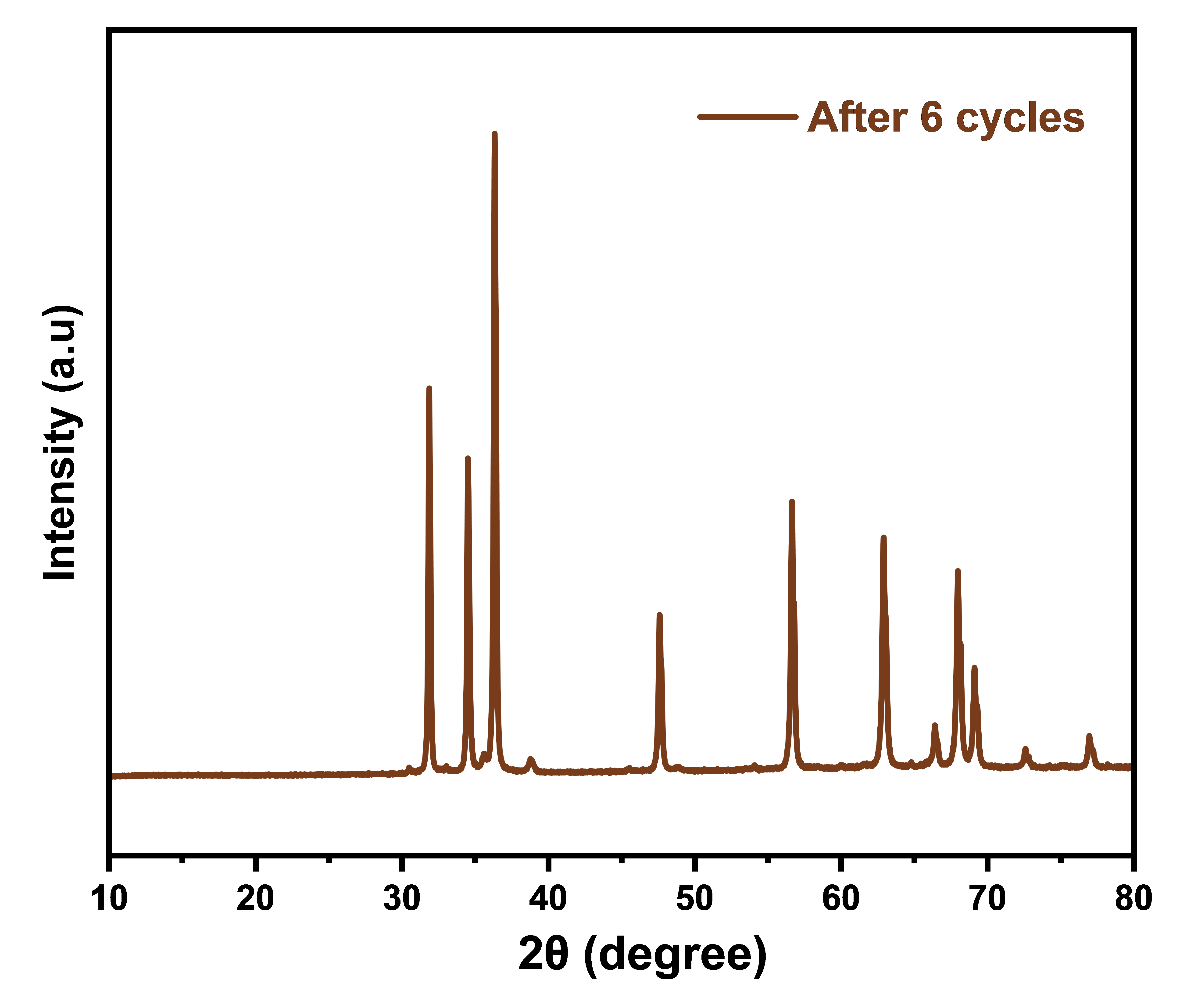


Fig.S4. XRD pattern after 6 cycles.


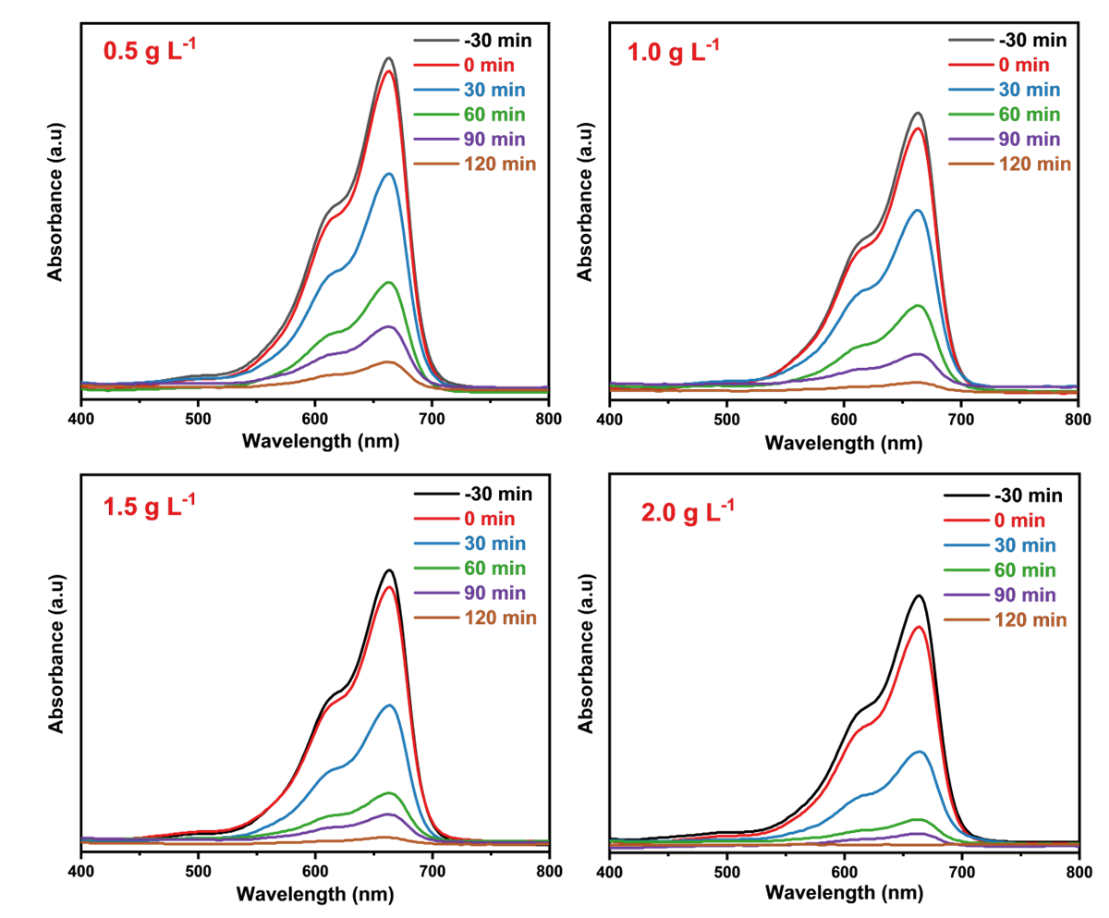


Fig. S5. UV-visible spectra of the effect of photocatalyst dose on the degradation of MB.


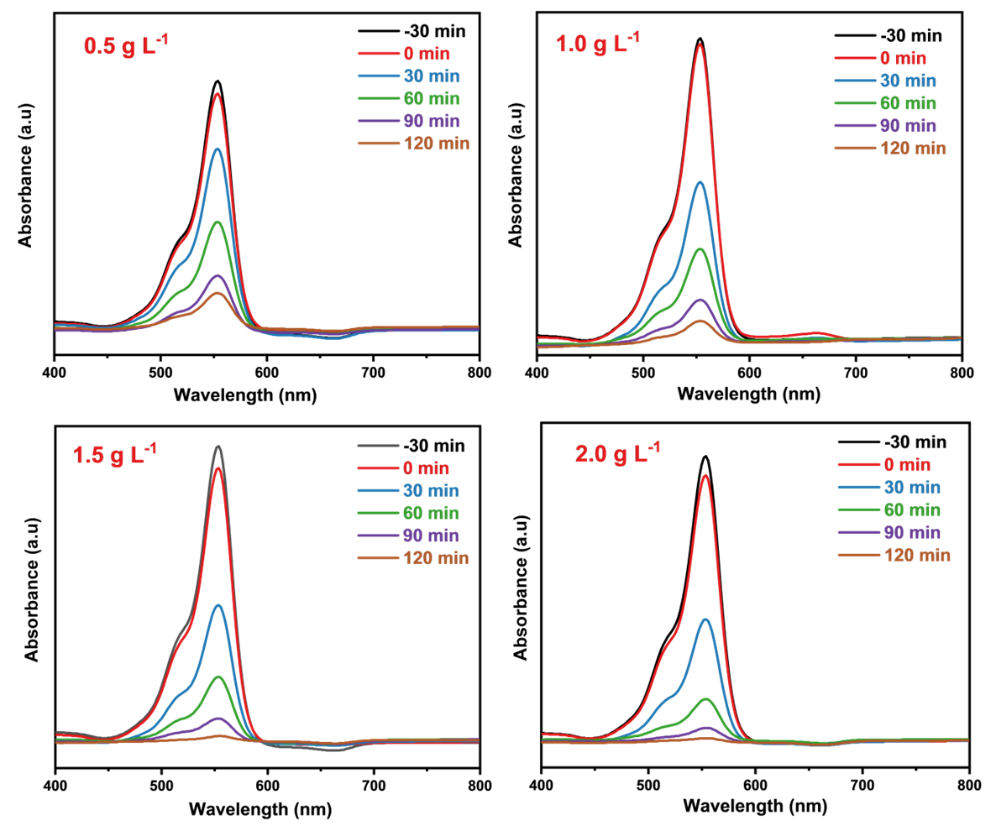


Fig. S6. UV-visible spectra of the effect of photocatalyst dose on the degradation of RhB.


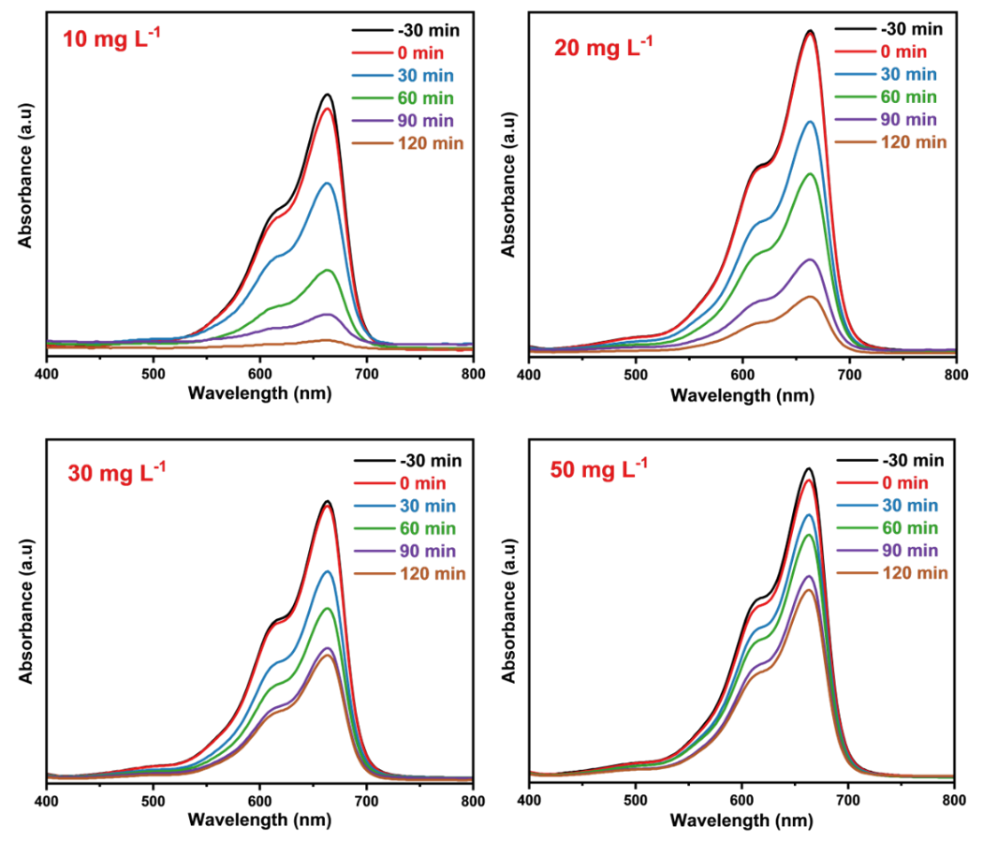


Fig. S7. UV-visible spectra of the effect of different concentrations of MB dye using CuO_0.25_@ZnO.


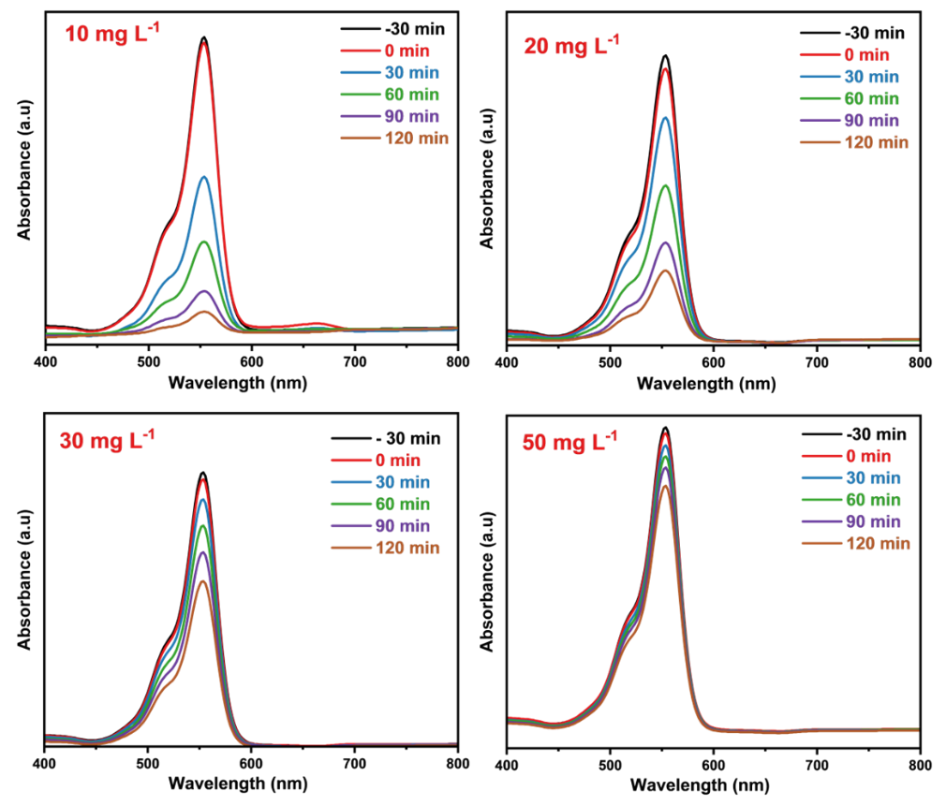


Fig. S8. UV-visible spectra of the effect of different concentrations of RhB dye using CuO_0.25_@ZnO.


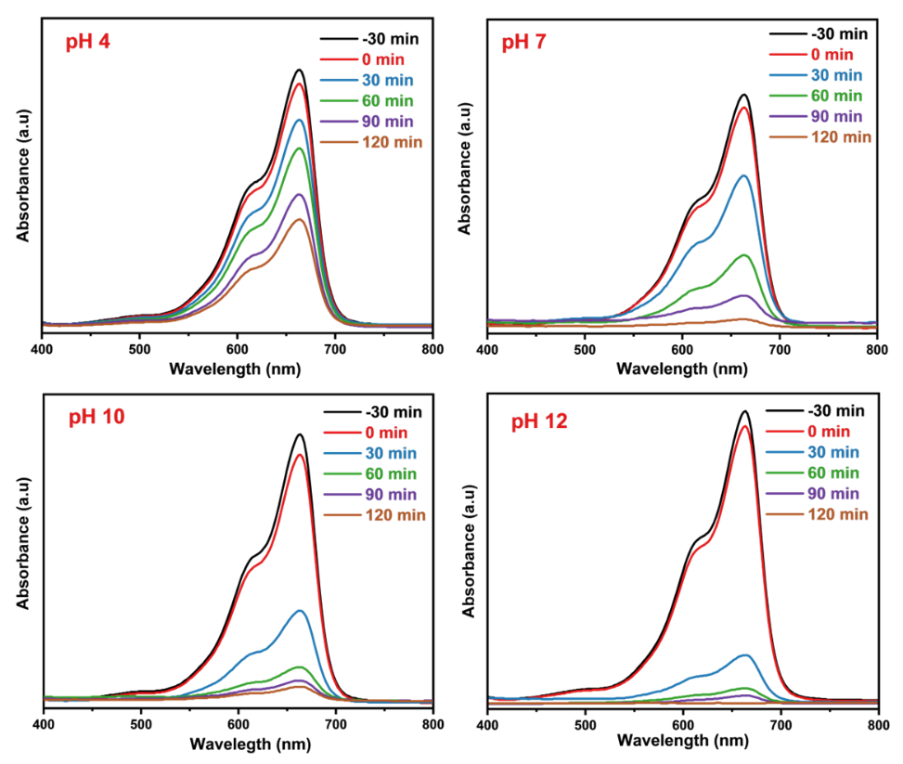


Fig. S9. UV-visible spectra of the effect of pH change on the degradation of MB dye using [CuO_0.25_@ZnO](mailto:CuO0.25@ZnO).


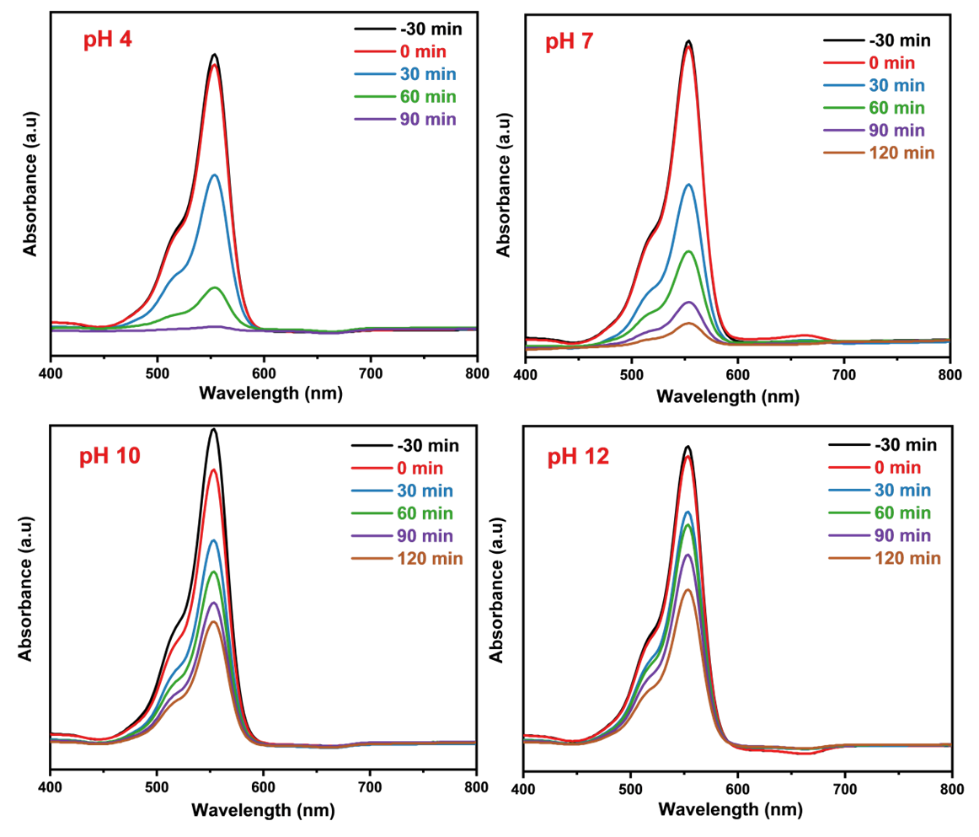


Fig.S10. UV-visible spectra of the effect of pH change on the degradation of RhB dye using [CuO_0.25_@ZnO](mailto:CuO0.25@ZnO).


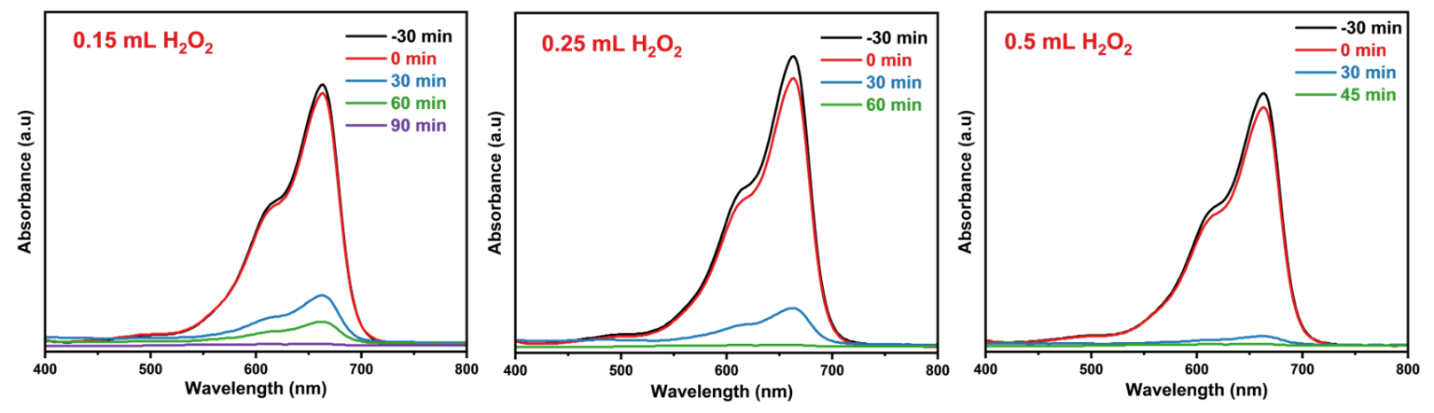
Fig. S11. UV-visible spectra of the effect of H_2_O_2_ concentrations on the photocatalytic degradation of MB dye using CuO_0.25_@ZnO nanocomposite.


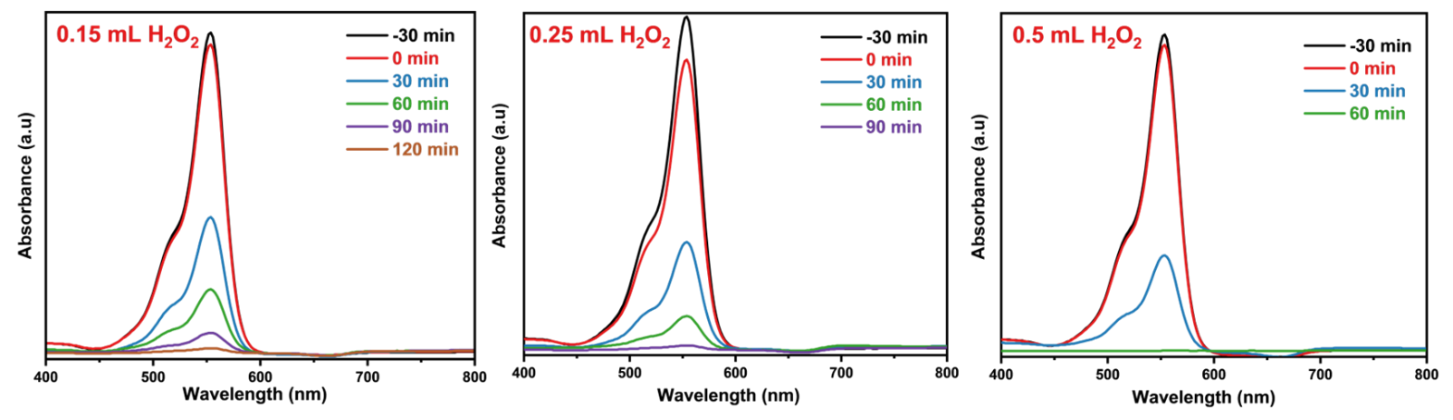


Fig. S12. UV-visible spectra of the effect of H_2_O_2_ concentrations on the photocatalytic degradation of RhB dye using CuO_0.25_@ZnO nanocomposite.


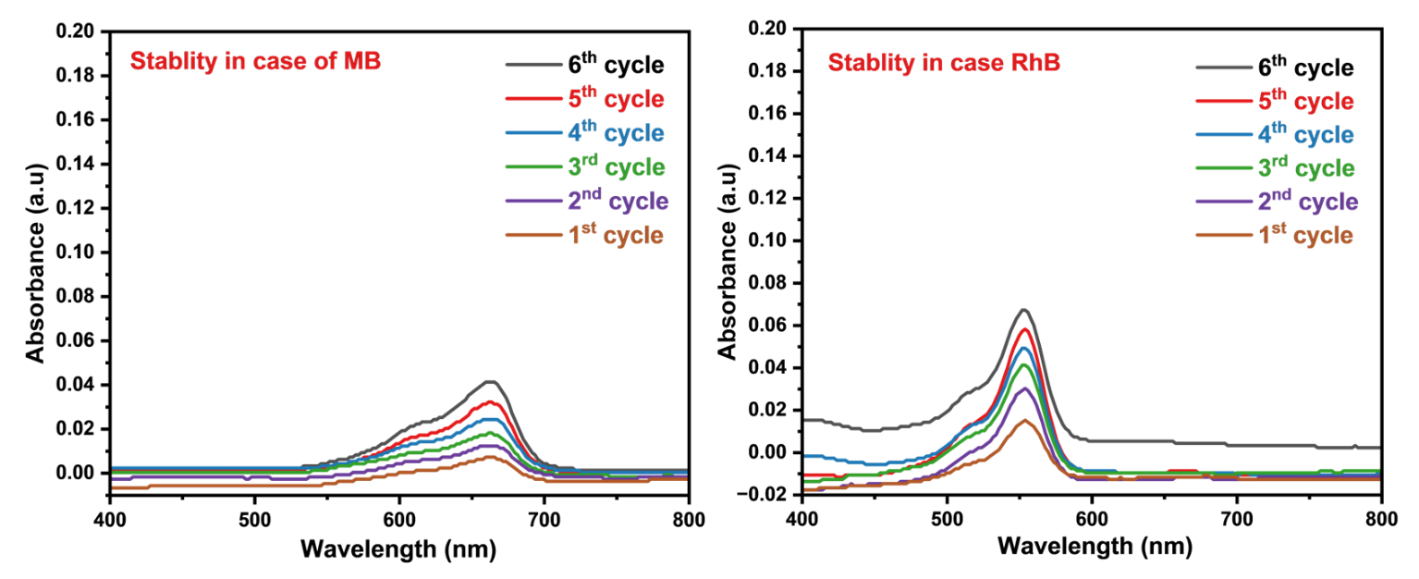
Fig. S13. UV-visible spectra of the reusability of CuO_0.25_@ZnO nanocomposite for photodegradation of MB and RhB dyes at 120 min over 6 cycles.

Fig. S14. Second-order kinetic model for different prepared nanocomposites (a) MB and (b) RhB dyes.


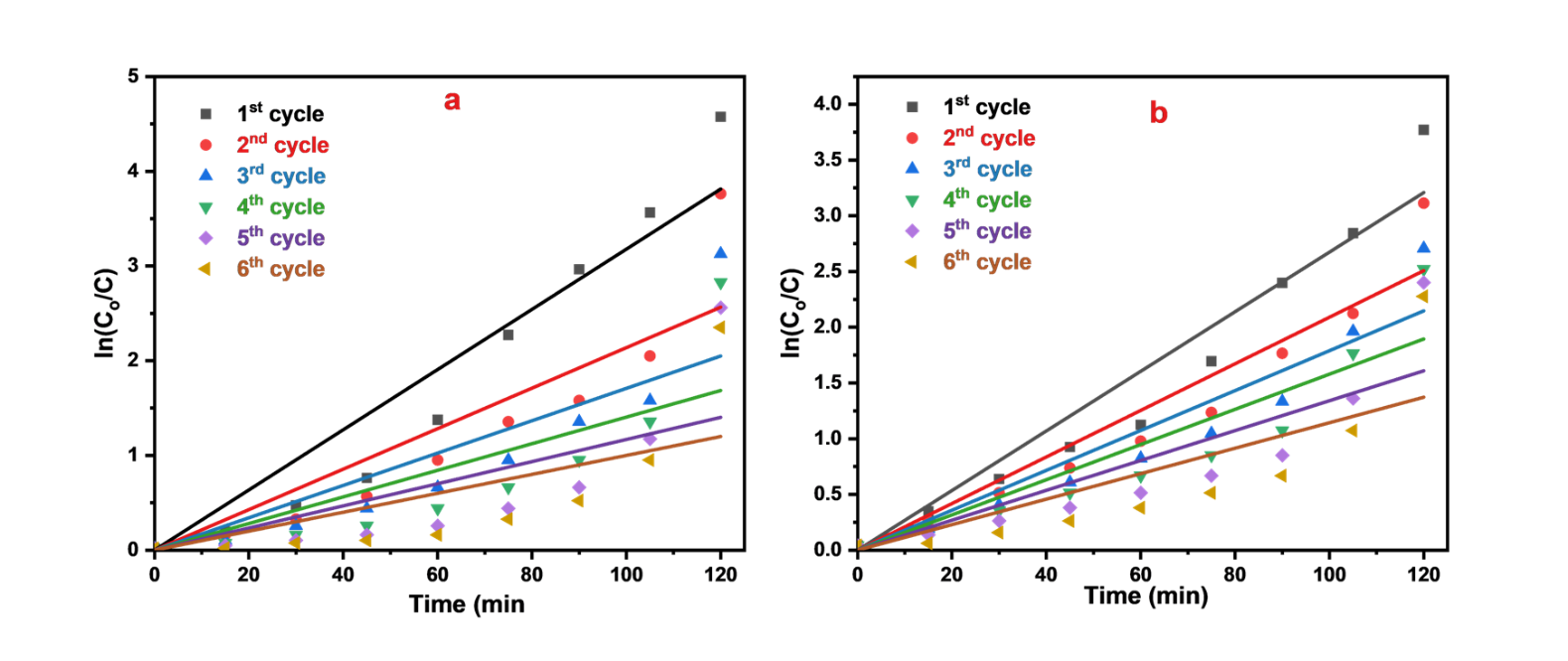


Fig. S15. First-order kinetic model of CuO_0.25_@ZnO nanocomposites for 6 successive cycles in case of (a) MB and (b) RhB dyes.

Table. S2. Cost-effectiveness of the fabricated photocatalyst CuO@ZnO

| **Material** | **Cost ($/g)** |
| --- | --- |
| PET plastic cups | ~ 0 |
| Electrical copper wires | ~ 0 |
| DMF | 0.143 |
| Methanol | 0.089 |
| Zinc acetate | 0.0257 |
| Sodium hydroxide | 0.0213 |
| CTAB | 0.0114 |
| Sum | 0.2904 |

Langmuir–Hinshelwood (L–H) mechanism, which is widely used to describe heterogeneous photocatalytic degradation processes occurring on catalyst surfaces.

In heterogeneous photocatalysis, the degradation reaction involves adsorption of dye molecules onto the catalyst surface, followed by surface redox reactions with photogenerated charge carriers and reactive oxygen species. According to the Langmuir–Hinshelwood model, when the initial dye concentration is relatively low, the surface coverage becomes proportional to dye concentration, and the kinetic expression can be simplified to pseudo-first-order behavior as follows:

$-\frac{\mathrm{dc}}{\mathrm{dt}}=\frac{k_{\mathrm{app}}C}{1+K_{e} C}$ (S1)

where; C represents the dye concentration (mg L^-1^), $k_{\mathrm{app}}$ is the reaction rate constant (mg L^-1^ min^-1^) and K_e_ is the equilibrium constant for the adsorption of the molecule on the catalyst surface (L mg ^-1^). The low concentration used in the experiments allows that $K_{e}C$ ≪ 1, and the kinetic expression can be simplified to pseudo-first-order behavior [1]. Therefore, the photocatalytic degradation kinetics were analyzed using the pseudo-first-order model, which is the low-concentration approximation of the Langmuir–Hinshelwood mechanism under dilute-dye conditions. In the present study, kinetic analysis was conducted at an initial concentration of 10 mg L^-1^ and showed a good linear fit to the pseudo-first-order model, confirming that the degradation process is governed by surface reaction kinetics under low-concentration conditions. At higher initial concentrations, degradation efficiency decreased due to limited active sites and light-scattering effects, making 10 mg L^-1^ the most suitable concentration for kinetic evaluation.

**References**

[1] N.G. Asenjo, R. Santamaría, C. Blanco, M. Granda, P. Álvarez, R. Menéndez, Correct use of the Langmuir–Hinshelwood equation for proving the absence of a synergy effect in the photocatalytic degradation of phenol on a suspended mixture of titania and activated carbon, Carbon, 55 (2013) 62-69.
